# Creating a Reliable Mass Spectral–Retention Time Library for All Ion Fragmentation-Based Metabolomics

**DOI:** 10.3390/metabo9110251

**Published:** 2019-10-26

**Authors:** Ipputa Tada, Hiroshi Tsugawa, Isabel Meister, Pei Zhang, Rie Shu, Riho Katsumi, Craig E. Wheelock, Masanori Arita, Romanas Chaleckis

**Affiliations:** 1 Department of Genetics, SOKENDAI (Graduate University for Advanced Studies), Shizuoka 411-8540, Japan; 2 RIKEN Center for Sustainable Resource Science, Kanagawa, Yokohama 230-0045, Japan; 3 RIKEN Center for Integrative Medical Sciences, Kanagawa, Yokohama 230-0045, Japan; 4 Division of Physiological Chemistry 2, Department of Medical Biochemistry and Biophysics, Karolinska Institutet, 171 77 Stockholm, Sweden; 5 Gunma University Initiative for Advanced Research (GIAR), Gunma University, Gunma 371-8510, Japan; 6 Center for Information Biology, National Institute of Genetics, Shizuoka 411-8540, Japan

**Keywords:** LC–MS, metabolomics, mass spectral deconvolution, chemical library, all ion fragmentation

## Abstract

Accurate metabolite identification remains one of the primary challenges in a metabolomics study. A reliable chemical spectral library increases the confidence in annotation, and the availability of raw and annotated data in public databases facilitates the transfer of Liquid chromatography coupled to mass spectrometry (LC–MS) methods across laboratories. Here, we illustrate how the combination of MS2 spectra, accurate mass, and retention time can improve the confidence of annotation and provide techniques to create a reliable library for all ion fragmentation (AIF) data with a focus on the characterization of the retention time. The resulting spectral library incorporates information on adducts and in-source fragmentation in AIF data, while noise peaks are effectively minimized through multiple deconvolution processes. We also report the development of the Mass Spectral LIbrary MAnager (MS-LIMA) tool to accelerate library sharing and transfer across laboratories. This library construction strategy improves the confidence in annotation for AIF data in LC–MS-based metabolomics and will facilitate the sharing of retention time and mass spectral data in the metabolomics community.

## 1. Introduction

Interest in the analysis of the metabolome has increased significantly due to its utility for understanding biological processes and for biomarker discovery [1]. Liquid chromatography coupled to mass spectrometry (LC–MS) is a widespread metabolomics method owing to its sensitivity, and its measurement strategies are broadly classified into targeted and nontargeted approaches [2]. Targeted approaches using LC–MS/MS offer increased selectivity and quantification [3]; however, they are by nature limited to the measurement of preselected compounds. Nontargeted metabolomics enables the discovery of unknown compounds; however, metabolite identification is a major bottleneck in data interpretation [4]. The criteria for compound identification were proposed more than a decade ago by the Metabolomics Standards Initiative (MSI) [5], in which four identification levels were described. To obtain the level-1 identification (most reliable), at least two orthogonal properties of the compound should match with those of an authentic standard. In LC–MS metabolomics, this criterion is often interpreted as an exact match of the peak feature in the measured sample and to the chemical standard by accurate mass (AM) and retention time (RT). However, these two properties may not be sufficient to reliably identify compounds due to co- or closely eluting compounds and RT fluctuations of certain chromatography techniques (e.g., HILIC).

**Figure 1:**
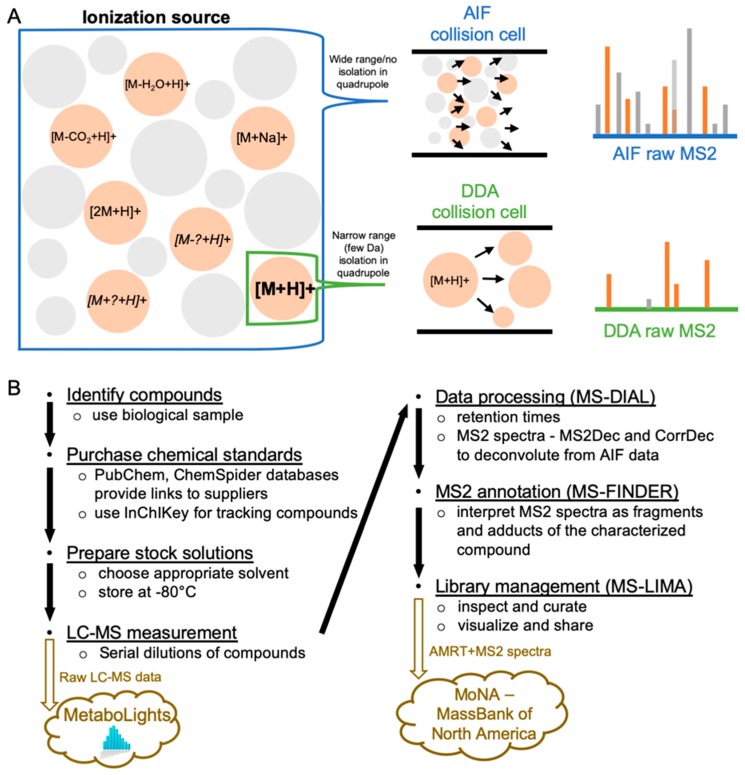
(**A**) Comparison of AIF (all ion fragmentation) and DDA (data dependent acquisition) MS2 spectra acquisition, (**B**) MS2 library construction workflow used in the current study.

To further increase the reliability of metabolite identification, MS2 spectra are used in addition to accurate mass and retention time (AMRT). MS2 spectra can be obtained from either data dependent acquisition (DDA) or data independent acquisition (DIA) [6]. In DDA, a narrow window of a few daltons or less is isolated around the precursor ion, and relatively clean MS2 spectra with a clear connection to their precursors are obtained [7]. However, MS2 information is obtained only for a fraction of all detected ions in a measured sample. In DIA, on the other hand, all ions are sent to the collision cell to obtain their cumulative MS2 spectra (Figure 1A); this means that MS2 information is collected for virtually all ions in the sample (provided that they are of sufficient abundance). DIA-based data such as AIF (all ion fragmentation), MS^E^, or SWATH (sequential windowed acquisition of all theoretical fragment ion mass spectra) [8] are therefore rich in content, but require spectral deconvolution. Towards this end, multiple software programs such as MS2Dec [9], MetDIA [10], and CorrDec [11] have been developed for interpretation of DIA-based data. In this process, there is little consensus on the treatment of spectra originating from identical compounds such as in-source fragmentation and different adducts [12]. In addition, peak intensities of MS2 spectra also depend on individual LC–MS instruments and measurement conditions [13]. Data analysis in DIA metabolomics is currently limited to the use of libraries constructed using DDA MS2 spectra without information on in-source fragmentation or multiple adduct types [14,15,16], or libraries with RT that are not suitable for the available measurement settings.

To address these difficulties and to provide a useful workflow for library construction, we demonstrate the creation of a reliable AMRT+MS2 library for LC–MS AIF metabolomics of hydrophilic compounds on a zic-HILIC column (Figure 1B). RT shifts were rigorously assessed using technical internal standards (tIS), and spectral deconvolution was fully exploited to obtain high-quality mass spectra for accurate metabolite annotation. A dedicated software tool was developed for comparing and sharing spectra in the NIST MSP format, named Mass Spectral LIbrary MAnager (MS-LIMA) [17]. Step-by-step tutorials are provided as supplemental materials for constructing (Tutorial 1) and application (Tutorial 2) of the AMRT+MS2 library on an AIF metabolomics dataset. While for simplicity the application in this work is limited to zic-HILIC chromatography, this approach is generally applicable to any chromatographic system.

## 2. Results and Discussion

### 2.1. Selection of Compounds for the Library

**Figure 2:**
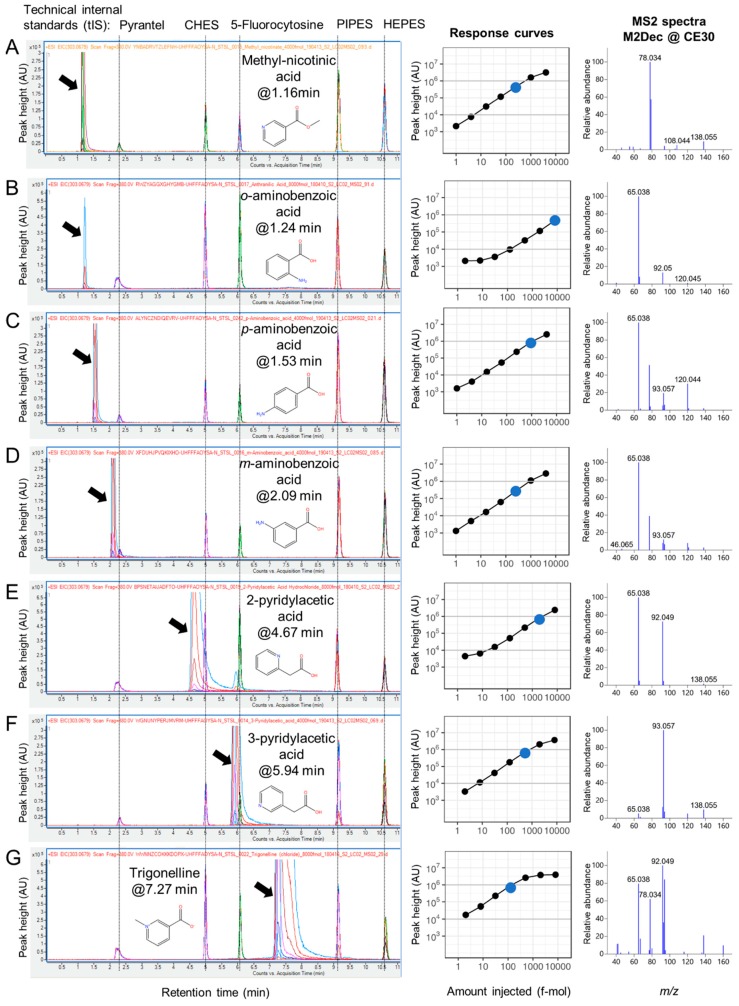
Retention time (RT) and response curve characterization of seven compounds with C_7_H_7_NO_2_ formula in positive ionization mode on zic-HILIC chromatography. Peaks of the characterized compounds are indicated by black arrows. The elution order of the methyl-nicotinic acid and aminobenzoates (**A**–**D**) was confirmed by the constant RTs of the tIS (technical internal standards). The analytical standard of 2-pyridylacetic acid (**E**) shows two peaks at 4.6 and 5.9 min, the later having the same RT as 3-pyridylacetic acid (see Figure S1) (**F**). Trigonelline (**G**) is detected at lower amounts than other compounds with the same formula. The shown MS2 spectra were deconvoluted using MS2Dec from the injection, indicated by a blue dot in the response curve.

Due to the need to perform multiple injections per compound, compound selection for inclusion in the library should be performed based upon likelihood of detection in authentic samples. We recommend establishing a list of compounds based upon feature annotation in the target sample matrix (e.g., pooled quality control samples, pilot study samples) [18,19]. Compounds can have multiple common names; for example, 5-pyrrolidone-2-carboxylic acid, pidolic acid, and pyroglutamic acid all designate the same chemical compound. In addition, identifiers from chemical databases such as KEGG [20], HMDB [21], ChEBI [22], PubChem [23], ChemSpider [24], or CAS numbers [25] do not necessarily contain all synonyms for a given compound. InChIKey is a universal and unique compound identifier developed under the auspices of IUPAC (International Union of Pure and Applied Chemistry) [26], which can be used to search for other identifiers automatically (for example, with the R webchem package [27] or Chemical Translation Service [28]). PubChem and ChemSpider provide comprehensive information on the compounds, including a list of vendors when available. Commercial compounds (this study Table S1) are often available as salts (e.g., trigonelline chloride), with varying degrees of purity. While composition and purity of the chemical standard is crucial for direct infusion, it is not critical when LC separation is used (Figure 2 and Figure S1).

Many plant- and food-based compounds are difficult to obtain commercially, as well as phase II metabolized forms (e.g., sulfates or glucuronides) of compounds other than drugs. While custom synthesis is an option, it is time-consuming, costly, and requires specific expertise [29]. When chemical standards are not available, the spectra of putatively annotated compounds in the samples can be used as an MSI level-2 or 3 compound library in order to reproduce consistent putative annotations across several studies.

### 2.2. LC–MS Acquisition of the Chemical Standard Spectra

When high-quality spectra are available, AIF data can be used to distinguish isobaric co- or closely eluting compounds [14,16,30]. However, compounds have different ionization efficiencies and response curves [31,32]. To produce a clean MS2 spectrum using MS2Dec [9], an appropriate amount for each compound should be injected into the LC–MS system. CorrDec [11] requires multiple samples, with varying levels of the target compound [11]. Therefore, multiple injections at different dilutions are necessary. Multiple injections also enable estimation of the detection and saturation limits for each compound.

In positive ionization mode, as used in the current study, compounds with positively charged nitrogen atoms (e.g., trigonelline or trimethylamino groups in betaines and carnitines) ionize very well (Figure 2). The detection limits for such compounds can be an order of magnitude lower (around 0.1 fmol) compared with the standard amino acids and nucleosides (1–10 fmol). On the other hand, compounds containing only carbon, oxygen and hydrogen (e.g., carboxylic acids) are often poorly detected in positive ionization, and negative ionization mode should therefore be used [33]. In addition, depending upon the compound, the molecular ion might not always be the major species [19]. For example, in this study the main ions of chenodeoxycholic and cholic acids in positive ionization mode are [M+H−2H_2_O]^+^ and [M+H−3H_2_O]^+^.

### 2.3. RT Characterization and Verification using Technical Internal Standards (tIS)

RT characterization initially appears to be straightforward, simply requiring notation of the elution time of the injected chemical standard on the LC–MS system. However, RT can fluctuate depending on many factors, including the LC–MS system setup, solvents, column batches, etc. [34]. For example, some HILIC columns are prone to fluctuations in RT even within the same system and sorbent batch (Figure S2), which can complicate method transfer across laboratories and decrease long-term consistency. The challenge of RT shifts can be illustrated using two isobaric compounds, valine and betaine. In Naz et al. [14], who employed the same zic-HILIC method and instrumentation as this study, valine and betaine eluted at 6.79 and 7.10 min, respectively, while in the current work, they eluted at 7.21 and 7.41 min, respectively. It is difficult to confidently identify these two compounds based solely on AMRT. The addition of MS2 spectra does not easily resolve this RT complication because low-molecular-mass metabolites with different structures may exhibit similar MS2 spectra as shown in Figure 2 for compounds with the formula C_7_H_7_NO_2_. RT characterization is necessary for reliable identification (see Section 2.6). Chemical standards may also contain impurities; for example, the peak of 2-pyridylacetic acid standard is separated by RT from 3-pyridylacetic acid (Figure 2E,F).

To address this issue, we include multiple tIS in each injection to check (1) the performance of the instrumentation (e.g., peak shape, intensity); and (2) RT shifts. In the GC–MS field, the Kovats retention indices have been used for decades to adjust the RT shifts. However, in the LC–MS field, there is no single set of widely adopted retention index standards [35,36,37]. RT standards were only recently proposed for HILIC chromatography [38]. A practical solution for selection of tIS is a mix of common metabolites or exogenous compounds as in this study, with RT spread across the elution profile. Alternative approaches to access RT shifts and tIS choices are summarized in Table S2.

To adjust the RT, we first obtain the reference RTs of the tIS from an authentic representative analysis (Table S3). Second, when processing each chemical standard data, their RTs are adjusted using the RTs of the tIS, based on a linear correction between each tIS. This is a relatively coarse correction, and other sophisticated approaches are available for larger deviations [39]. Information on the fluctuations of the tIS RTs from the library construction can be used when setting RT tolerance for compound identification in a dataset.

For the five tIS (Figure 2) used in this study, we observed RT deviations <0.55 min from average (Figure S3) and coefficient of variation (CV) across the seven injections of the 140 compounds in most cases <10% (Table S4). We observed ion suppression when a tIS coeluted with a characterized compound (e.g., fluorocytosine coeluted with norvaline betaine, resulting in ion suppression at 6.10 and 6.16 min, respectively).

Currently the AMRT libraries can only be used for MSI level-1 annotation if generated in the same laboratory under identical experimental conditions. We demonstrate here that, in reality, experimental conditions fluctuate over time, even in the same laboratory on the same instrument (e.g., solvent, column production batches), greatly affecting the RT precision. Therefore, in current practice, untargeted metabolomics studies should only report MSI level-2 annotations, unless all standard compounds are simultaneously analyzed within the same analytical batch/study. However, the use of measurable parameters such as RT deviations of the tIS should enable researchers to assess whether the library is suitable for the AMRT MSI level-1 annotations of a dataset.

### 2.4. MS2 Spectra Deconvolution and Annotation of Major Ions Using AIF Data

A high-quality library requires annotation of reliable product ions in MS2 spectra of the chemical standards. Comparison of the annotated compound MS2 spectra enables the search for compound-specific fragment ions. In the case of complex AIF data from biological samples, such compound-specific ions enable quantification of coeluting compounds such as threonine/homoserine [14] methylxanthines [30], or leucine/isoleucine [16]. In principle, DDA MS2 spectra can be used to identify such compound-specific ions, however, for example, DDA MS2 spectra obtained by direct infusion do not account for the in-source fragmentation as well as may contain peaks from isobaric impurities. Therefore, we recommend using annotated AIF MS2 spectra obtained from the characterization of chemical standard dilution series.

We used two deconvolution methods based on different concepts. MS2Dec [9] applies a least square regression method to consider the difference of liquid chromatographic peak tops, while CorrDec [11] calculates the Pearson’s correlation among multiple samples to identify correlated MS2 peaks with the precursor. In other words, MS2Dec and CorrDec consider different information: ion intensity over RT in MS2Dec, and ion intensity across samples in CorrDec.

From the dilution series, a representative sample (at nonsaturated ion intensity corresponding to 10^4^–10^6^ AU, with the instrumentation and settings used in this study) was selected for each chemical standard. For all 140 compounds, raw MS2 spectra were obtained at 0, 10, and 30 eV collision energies. The median number of peaks in raw MS2 spectra were 52 (0 eV), 91 (10 eV), and 128 (30 eV) after removing small peaks with <1% relative ion intensities. Spectra were then deconvoluted using both MS2Dec and CorrDec. CorrDec was able to generate deconvoluted MS2 spectra for 132 of the 140 compounds, with eight compounds not fulfilling the CorrDec criteria (at least four spectra of each compound have to be above the noise level). The two deconvolution methods produced similar spectra (the median dot product similarity: 81.3%), although their concepts and calculation methods are fundamentally different. The median number of peaks in MS2Dec spectra were 8, 15, 19, and in CorrDec spectra, 10, 19, 22 at 0, 10, 30 eV, respectively.

After deconvolution, MS2 peaks in each spectrum were annotated using the fragment annotation method implemented in MS-FINDER [40]. The MS-FINDER version 3.22 or later can estimate not only formula and substructure, but also isotopic ions and different adduct types of MS2 peaks from AIF data (AIF MS2 spectra may include different adduct types due to multiple precursors as explained in the Introduction). Nonannotated peaks were removed from the spectra, and the median number of removed peaks was four in both MS2Dec and CorrDec.

**Figure 3:**
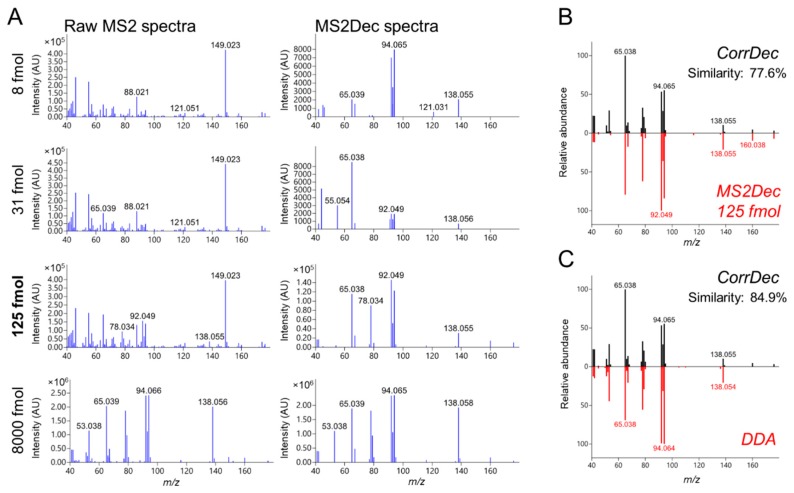
Deconvolution of trigonelline (C_7_H_7_NO_2_, monoisotopic mass 137.0477) MS2 spectra from AIF data at 30 eV. (**A**) Raw trigonelline AIF spectra contain multiple noise peaks (left column), compared with MS2 spectra deconvoluted by MS2Dec (right column), especially when lower amounts were injected. (**B**) MS2Dec and CorrDec yield similar MS2 spectra. (**C**) Comparison between CorrDec and DDA MS2 spectra acquired in house at 30 eV (MoNa ID: MoNA011431) confirms the solid MS2 deconvolution from the AIF data. Similarity reported as the dot product.

We detail our approach using the example of trigonelline, a betaine-type compound, made by plants and often detected in human biofluids [29,41]. Trigonelline ionizes well, and a relatively low amount of 125 fmol was sufficient to obtain a high (ion intensity: 907588), but nonsaturated, signal (Figure 2G). In the raw MS2 spectra at 30 eV (Figure 3A left column), the difference in the fragment patterns among the dilution series was observed. There was a common peak (149.022 *m/z*) detected in even the lowest concentration, which was most likely chemical noise (possible formula: C_8_H_5_O_3,_ corresponding to the common contaminant phthalic acid [M+H−H_2_O]^+^ ion [42,43]). The MS2Dec spectra (Figure 3A, right column) were similar (the median similarity of all MS2Dec pairwise comparisons: 90.8%) over the dilution series. The only exception was the 31 fmol sample, whose base peak was 65.038 *m/z* (the median similarity between MS2Dec 31 fmol spectra and the other MS2Dec spectra: 49.0%); however, this peak was a fragment of trigonelline in combination with noise. A comparison of trigonelline’s raw spectrum (Figure 3A, left column) to MS2Dec spectra (Figure 3A, right column) shows that deconvolution is indeed effective. The CorrDec spectra were generated using seven raw MS2 spectra and compared to representative MS2Dec spectrum, showing a good match (Figure 3B). In both spectra, the primary adduct type observed was [M+H]^+^ (138.055 *m/z*). Additionally, [M+Na]^+^ (160.038 *m/z*) and [M+K]^+^ (176.012 *m/z*) were also detected. The sodium and potassium adducts probably originate from the chemical standard, purchased as trigonelline chloride (see Table S1). To confirm the reliability of trigonelline’s MS2Dec and CorrDec deconvoluted spectra, they were compared with the DDA MS2 spectra measured in house (Figure 3C). Although raw AIF MS2 spectra are noisy, the deconvoluted and curated MS2 spectra were well matched with the DDA MS2 spectrum. MS2 spectra deconvoluted from AIF data offer advantages relative to DDA MS2 spectra, including good coverage of isotopic patterns and inclusion of the adducts relevant to the LC method used in the acquisition (Figure 3C).

### 2.5. Confirmation and Curation of MS2 Spectra using MS-LIMA

**Figure 4:**
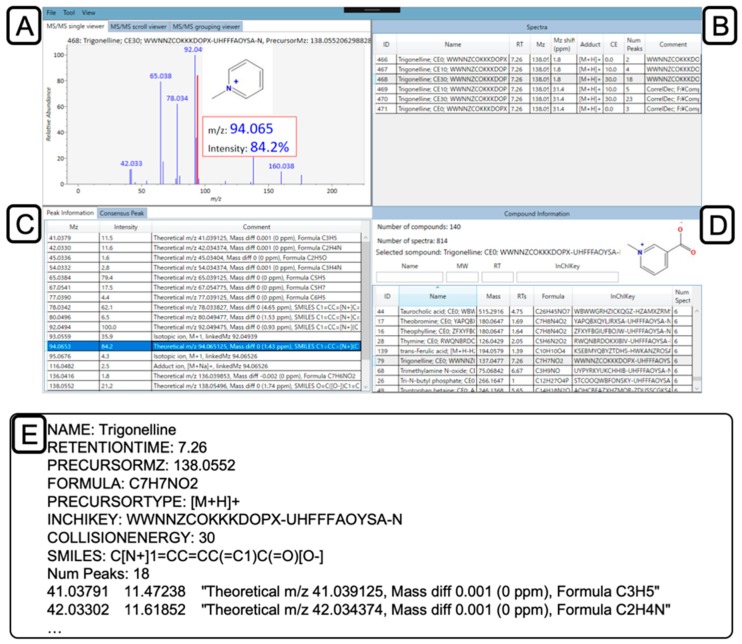
MS library organization and editing with MS-LIMA. (**A**) Visualization of MS spectrum with (**B**) editable annotations from MS-FINDER for each peak. (**C**) Available MS spectra for (**D**) a selected compound in loaded AMRT+MS2 library. (**E**) For MS-LIMA libraries, we recommend to include the following lines for each record with trigonelline as an example.

An open-source library editor, MS-LIMA, was developed to visualize, manage, and curate mass spectral libraries. The main window of MS-LIMA is shown in Figure 4, demonstrating the display following opening of the library described above and selecting the peak at 94.065 *m/z* originating from the trigonelline spectrum at 30 eV. MS-LIMA supports MassBank, MGF, and many subtypes of MSP formats [44] from multiple institutes and databases, such as RIKEN [45], MoNA [46], and NIST [47].

After opening library files, MS-LIMA groups compound spectra based upon the InChIKey or the first 14 characters of the InChIKey corresponding to the molecular skeleton [26]. This makes it easy to compare and assess MS2 spectra originating from the same compound. In the grouping process, MS-LIMA checks all MS2 records from the same compound to ascertain whether they share an identical formula and similar RT (<1 min difference as default). This limits the possibility that the given MSP files contain RTs from different LC methods. MS-LIMA also supports MS2 annotated peaks by MS-FINDER version 3.22 or later and visualization of the substructure for the selected peak (Figure 4C). To curate spectra, users can check precursor *m/z* differences and modify all information in the library. Also, MS-LIMA has various functions to manage and curate the library, including MS2 spectra comparison between two libraries, making a consensus spectrum of a compound, calculating the frequency of product ions among library, automatically saving, exporting spectrum as several formats, and replacing metadata based on InChIKey (see GitHub repository for details [17]). Moreover, because it is open-source, anyone can contribute to the development of MS-LIMA to support additional formats or add new functions.

With the MS-LIMA version 1.52, we examined 814 MS2 spectra (140 compounds) exported from MS-FINDER: compared the precursor *m/z* difference with theoretical *m/z*, confirmed adduct type and collision energies, and removed nonannotated MS2 peaks. The experimental precursor *m/z* was replaced with the theoretical precursor *m/z*, because the characterized compounds were known and theoretical precursor *m/z* values should be used in the mass spectral search to calculate the mass accuracy. The original experimental *m/z* values were stored, because it is also important to know the mass accuracy of spectral records. For example, the information of mass accuracy is necessary for structure elucidation tools such as MS-FINDER [40] and CSI:FingerID [48]. Although the MS1 mass accuracy cannot directly be transferred to the MS2 mass accuracy, the experimental precursor *m/z* value is a criterion to access accuracy in MS1 and MS2 spectra. Finally, we modified and added metadata, including SMILES, InChI, spectrum type, instrument, instrument type, chromatography, author, and license. As described in the methods section, raw data has been deposited to the EMBL-EBI MetaboLights repository [49] with the identifier MTBLS816, the MS2 spectral library was submitted to MoNA [46], and the RTs of compounds were also deposited at PredRet database [50], with the benefit of predicting RTs for uncharacterized compounds by mapping between multiple chromatographic systems. Raw data and MS spectra can also be deposited in other repositories (e.g., Metabolomics Workbench [51] and GNPS [52]). In this study, we used MS-DIAL and MS-FINDER to obtain the MS spectra from the AIF data; however, alternative workflows can be created using other available tools including MZmine [53], XCMS [54], CAMERA [55], RAMClust [56], CliqueMS [57], mzCloud [58], MetFrag [59], and CSI:FingerID [48]. In the era of open science, sharing and obtaining feedback on the MS2 libraries is necessary for improving the quality as well as for developing the metabolomics community.

### 2.6. Library Application for Human Urine Study and Limitations

**Figure 5:**
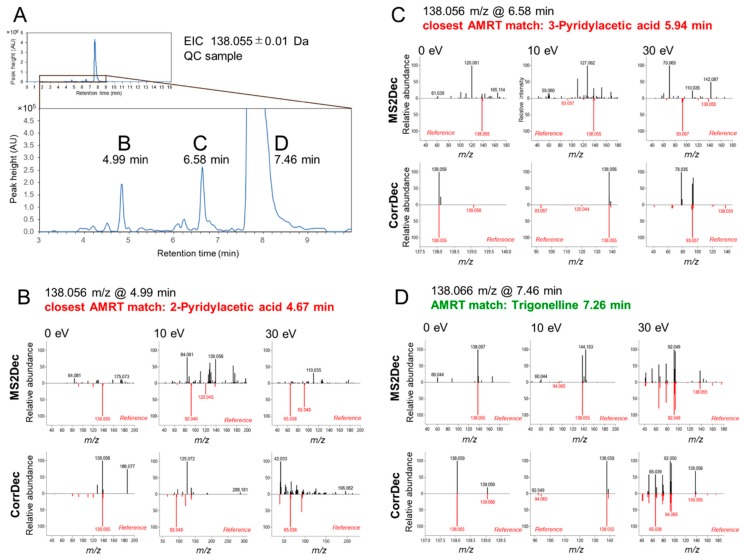
Application of the AMRT+MS2 library to urine metabolomics data acquired in positive ionization mode on a zic-HILIC column. (**A**) Extracted ion chromatogram of *m/z* 138.055 ± 0.01 Da (corresponding to [C_7_H_7_NO_2_+H]^+^) from a quality control (QC) sample. Two peaks at (**B**) 4.99 min and (**C**) 6.58 min have AMRT matches within 0.7 min, but poor MS2 match despite relative high abundance. A peak at 7.46 min (**D**) despite the mass shift due to high abundance could unequivocally be identified as trigonelline based on the AMRT+MS2 match (trigonelline was not spiked into the sample or known a priori to be present in the samples).

A 224-sample urinary metabolomics study measured by AIF was used for library assessment. The dataset has been deposited to the EMBL-EBI MetaboLights repository [49] with the identifier MTBLS816. To highlight the benefits of our library, we focused on the particular *m/z* window, 138.055 ± 0.01, which could correspond to [C_7_H_7_NO_2_+H]^+^; the details and additional examples are provided in the supplemental compound identification in the LC–MS AIF data tutorial (Tutorial 2). Based upon AMRT match only, which qualifies for MSI level-1, three features had plausible matches in our library (Figure 5A). With respect to MS2, two features at 4.99 min and 6.58 min did not match to any spectra in spite of relatively high ion abundance (Figure 5B,C). In contrast, a peak at 7.46 min could be identified as trigonelline, based on not only the AMRT, but also the MS2 match (Figure 5D). Therefore, we consider the two peaks at 4.99 and 6.58 min as adduct ions, in-source fragments, or unknown compounds. Due to RT fluctuations in HILIC chromatography, relatively large tolerances are used at the cost of reliable identification, and it is essential to use MS2 matching whenever possible to ensure accurate annotation.

Although we highlighted the advantages of the created library, there are limitations. The library spectra were obtained from our LC–MS platform (Agilent Technologies, Santa Clara, CA, USA), and the spectra will most likely differ on platforms from other MS vendors with different ionization configurations. The set of tIS was chosen for our zic-HILIC method using positive ionization mode, and a different set may offer improved performance for a different combination of chromatography system, sample type, and ionization mode. For example, positive ionization mode is suitable for the urine study due to its efficient ionization of nitrogen-containing metabolites. However, negative ionization mode will require a different set of tIS, while reversed phase would yet again require a unique set of tIS. In this sense, it is difficult to assess the efficiency of our library only from a single study. However, the methodology introduced here is clearly transferrable, and there is a need to standardize this process within the metabolomics community. We emphasize the importance of RT characterization and extensive curation of spectra, and MS-LIMA has been useful for our workflow to create the library.

## 3. Conclusions

Reliable AMRT+MS2 libraries are needed in order to confidently annotate metabolites in LC–MS data. Herein, we describe a workflow to obtain AM, RT, and MS2 for a given compound using the AIF data acquisition method and provide practical recommendations for library development. In order to facilitate library curation and visualization, we developed the spectra manager MS-LIMA. The construction of high-quality, open-access libraries makes compound annotations more transparent, reliable, and transferable to the broader community.

## 4. Materials and Methods

### 4.1. Materials

Water, acetonitrile, methanol, and isopropanol used for the LC–MS analysis and sample preparation were of LC–MS grade and purchased from Wako (Osaka, Japan). Chemical compounds were purchased from the vendors specified in Table S1.

### 4.2. Compound Preparation for Analysis

A stock solution (1–10 mM) for each chemical standard (Table S1) was prepared in water, methanol, acetonitrile, or other suitable solvent and stored at −80 °C. For the LC–MS characterization, seven 4-fold serial dilutions from 4.0–0.001 µM were prepared for each compound in acetonitrile containing tIS (Tables S1 and S3). An Agilent Bravo liquid handling system (Agilent Technologies, Santa Clara, CA, USA) with 96-well 0.2 mL PCR plates (PCR-96-MJ, BMBio, Tokyo, Japan) was employed to automate the serial dilutions. Pierceable seals 4Ti-0531 (4titude, Wotton, UK) were used to seal the plates for 4 s at 185 °C, using a PX1 heat sealer (Bio-Rad, Hercules, CA, USA). The plates were stored at 4 °C until measurement by LC–MS. See also tutorial chemical standard characterization using LC–MS AIF data (section “Handling of chemical standards and LC–MS measurements”).

### 4.3. Data Acquisition

LC–MS measurements in AIF mode were performed as described previously [14,30], with LC and MS settings detailed in Tables S5 and S6 respectively. In short, metabolites were separated on a 15 min gradient using a zic-HILIC column (100 × 2.1 mm, 3.5 μm particle size; Merck, Darmstadt, Germany) with acidified water and acetonitrile. Data were acquired in positive ionization mode on an Agilent 6550 Q-TOF-MS system (Agilent Technologies, Santa Clara, CA, USA), with a mass range of 40−1200 *m/z* in AIF mode, with three alternating collision energies (full scan, 10, and 30 eV). The data acquisition rate was 6 scans/s. One or two microliters of the solution were injected into the LC–MS system, corresponding to 1–8000 fmol. Solutions were injected from the lowest to the highest concentration, with a blank sample between each compound. The LC system was conditioned with several injections before each LC–MS sequence, and in each injection, a 7 min re-equilibration step was implemented after the gradient to maintain stable RTs.

### 4.4. Data Analysis

Data files were converted to mzML format using ProteoWizard version 3.0 [60] and processed in MS-DIAL [9] version 3.66 to obtain RT and MS2 spectra using MS2Dec and CorrDec deconvolution algorithms. The CorrDec function is implemented in the MS-DIAL (version 3.32 or later), which is freely available [61]. Next, peaks in each MS2 spectra were annotated in MS-FINDER [40] version 3.22 and exported in NIST MSP format. Detailed settings of MS-DIAL and MS-FINDER can be found in Tables S3 and S7–S9. See also tutorial chemical standard characterization using LC–MS AIF data (Tutorial 1, sections “Deconvolution MS2 spectra in MS-DIAL” and “Annotation of MS fragments in MS-FINDER“).

In order to curate and maintain the mass spectral libraries, we developed MS-LIMA software (open source, available on GitHub MS-LIMA project [17]). The library presented here was curated using MS-LIMA version 1.52 in the following manner: we replaced the experimental precursor *m/z* with the theoretical values (because the identity of the compound being characterized was known in each case) and kept only the peaks with the MS-FINDER formula annotation (isotopes, fragments, adducts) in the mass spectra. See also tutorial chemical standard characterization using LC–MS AIF data (Tutorial 1, section “Library assembly and curation in MS-LIMA”).

### 4.5. Data Availability

The dataset has been deposited to the EMBL-EBI MetaboLights repository [49] with the identifier MTBLS1040. MS2 spectra were submitted to RIKEN PRIMe website [45] and MoNA (MassBank of North America [46]) with the tags: “zicHILIC_POS_KI-GIAR”, “Agilent_6550_Q-TOF_AIF”. RT were submitted to PredRet [50] and assigned to the chromatography named “KI_GIAR_zic_HILIC_pH2_7”, containing also records from a previous publication [14].

## Supplementary Materials

The following are available online at https://www.mdpi.com/2218-1989/9/11/251/s1, Tutorial 1: Chemical standard characterization using LC–MS AIF data for AMRT+MS2 library, Tutorial 2: Compound identification in LC–MS AIF data using AMRT+MS2 library, Figure S1: Identification of the contaminant peak as 3-pyridylacetic acid in the chemical standard of 2-pyridylacetic acid. EIC for the [M+H]^+^ molecular ion of the pyridylacetic acid (**A**) shows a major peak at 4.7 min and a minor peak at 5.9 min (4.7% intensity of the major peak, RT matches 3-pyridylacetic acid). MS2 match (dot score) of the minor peak (5.9 min) MS2Dec deconvoluted spectra to the 2-pyridylacetic acid reference spectra (**B**) is worse than the match to 3-pyridylacetic acid reference spectra (**C**). According to the certificate of analysis, the purity of the 2-pyridylacetic acid standard was 97% by NMR, therefore we conclude that the minor peak at 5.9 min can be interpreted as 3-pyridylacetic acid contamination in the chemical standard of the 2-pyridylacetic acid, Figure S2: Same model HILIC column as used in this study (Merck SeQuant ZIC HILIC 2.1 × 100 mm, 3.5 µm particle size) shows RT fluctuations under identical conditions. One microliter of methanol solution containing (**A**) 0.5 μM CHES, (**B**) 0.1 μM Fluorocytosine, (**C**) 1.0 μM PIPES, (**D**) 1.0 μM HEPES, (**E**) 2.0 μM L-Histidine-15N3 was injected three or two times onto the conditioned zic-HILIC columns with same solvents and LC–MS system. Four columns were from the same sorbent batch (serial numbers: 912323, 912371, 912383, 912400), while one was from different one (649173), Figure S3: RT corrections for all 140 library compounds (seven injections per compound) show (**A**) the deviation of <0.55 min for each technical internal standard from the initial settings (**B**), Table S1: Authentic chemical standards, Table S2: Alternative approaches to access RT shifts and tIS choices, Table S3: Technical internal standard settings for retention time normalization, Table S4: CVs of the tIS peak intensities in the 140 compound data, Table S5: HILIC liquid chromatography settings, Table S6: Mass spectrometry parameters in positive ionization mode, Table S7: MS-DIAL console project settings, Table S8: MS-DIAL experiment file for multiple collision energy mode, Table S9: MS-FINDER parameter settings.
