# Supplementary material for: Creating a Reliable Mass Spectral–Retention Time Library for All Ion Fragmentation-Based Metabolomics
Source: Metabolites. 2019 Oct 26;9(11):251. doi: 10.3390/metabo9110251 (PMC6918128; doi:10.3390/metabo9110251)
Supplement: Supplementary file 1 [file metabolites-09-00251-s001.zip › supplementary materials/Tutorial 1. Chemical standard characterization using LC-MS AIF data for AMRT MS2 library.pdf]

Chemical standard characterization using AIF acquisition to obtain deconvoluted MS2 spectra and RT for building an AMRT+MS2 library

Content

1. OVERVIEW 2

2. HANDLING OF CHEMICAL STANDARDS AND LC–MS MEASUREMENTS 2

3. DECONVOLUTION MS2 SPECTRA IN MS-DIAL 4

4. ANNOTATION OF MS FRAGMENTS IN MS-FINDER 11

5. LIBRARY ASSEMBLY AND CURATION IN MS-LIMA 12

If used in data processing for publication, please cite:
Tada, I.; Tsugawa, H.; Meister, I.; Zhang, P.; Shu, R.; Katsumi, R.; Wheelock, C.E.; Arita, M., Chaleckis R. Creating a Reliable Mass Spectral–Retention Time Library for All Ion Fragmentation-Based Metabolomics. *Metabolites* **2019**

Contact: Romanas Chaleckis (romcha@gunma-u.ac.jp), International Open Laboratory (Karolinska Institutet) at Gunma Initiative for Advanced Research (GIAR), Japan

1. Overview

This tutorial describes the characterization of chemical standards for metabolomics using an all ion fragmentation (AIF) LC–MS method to obtain deconvoluted MS2 spectra (MS2Dec and CorrDec) and retention time to create an AMRT+MS2 library. This library can be used for MSI level-1 metabolite annotations of metabolomics datasets acquired using the same LC–MS method. The flowchart of the workflow is provided in Figure 1.

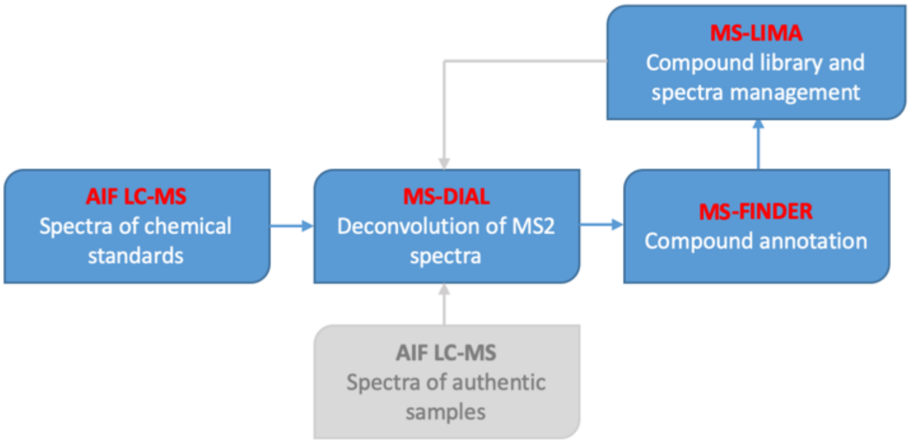

Figure 1. Flowchart of the workflow for constructing AMRT+MS2 library.

The protocol consists of the following steps below:

- Handling of chemical standards and LC–MS measurements
- Deconvolution MS2 spectra in MS-DIAL
- Annotation of MS2 fragments in MS-FINDER
- Library assembly and curation in MS-LIMA

Example LC–MS files can be obtained from Metabolights repository, study MTBLS1040 (https://www.ebi.ac.uk/metabolights/MTBLS1040).

Software is available for download:

-MS-DIAL (version 3.66 or later) (http://prime.psc.riken.jp/Metabolomics_Software/MS-DIAL/index2.html)

-MS-FINDER (version 3.22 or later) (http://prime.psc.riken.jp/Metabolomics_Software/MS-FINDER/index2.html)

-MS-LIMA (version 1.52 or later) (https://github.com/tipputa/MS-LIMA-Standard)

2. Handling of chemical standards and LC-MS measurements

Compounds are purchased from chemical vendors and are best managed in a database or excel sheet. To have a reliable record, (**1**) chemical name, (**2**) company and (**3**) catalogue number, and (**4**) **InChIKey of metabolite** should be recorded. **InChIKey of the metabolite** provides the connection with metabolomics analysis. Using the InChIKey, other identifiers can be looked up in databases (PubChem, ChemSpider), Chemical Translation Service (https://cts.fiehnlab.ucdavis.edu/), or webchem package in R.

NOTE: InChIKeys are different for a metabolite and its salt (compounds often sold as salts (e.g., trigonelline chloride, sugars derivatives, and sugar-phosphates as lithium salts) or mixtures (e.g., pyrantel pamoate)); also care should be taken with tautomeric compounds (e.g., sugars) as different tautomers have different InChIKeys, and where possible use the noncharged molecule InChIKey.

Weigh several mg of the compound in a 15 or 50 mL falcon tube and dissolve to the target 1–10 mM concentration in a suitable solvent (MeOH, water, etc.) to obtain the stock solution. Prepare aliquots (1 mL into two 1.5 ml vials) for long-term storage (e.g., –20°C or –80°C).

Stock solutions of the chemical standards are diluted using solvent containing technical internal standards (tIS) to keep track of instrument performance (injection errors, eventual retention time shifts, etc.), as shown in Table 1. For convenience and time efficiency, compounds can be characterized on 96-well plates: the first row contains up to 12 chemical standards diluted from the stock solutions (1–10 mM) to 40 µM with the tIS solvent mix and subsequent rows 7 serial dilutions.

In Table 1, we show a pipetting scheme for a 96-well plate: pipette 70 µl of the 40 µM solutions of the compounds into row A. Transfer the tIS solvent mix using a multichannel pipette: 135 µL to row B, 120 µL to rows C–H. Next, perform a 10-fold dilution by mixing row A, and transfer 15 µL to row B. Then, proceed with 4-fold dilutions by transferring 40 µL after mixing from row B to row C, and repeat until row H. Seal the plate and proceed with LC–MS measurements in AIF mode.

CAUTION: Starting concentration and dilution steps might need to be adjusted depending on the LC–MS method and system. Different structures of a compound may require a different collision energy for optimal MS2 results. A balance should be found between the number of collision energies and sensitivity/spectra acquisition time.

Table 1. Example chemical standard dilutions and the amounts injected in the LC–MS system.

Row	Concentration µM	Dilution factor	Amount in 1 µl injection fmol
A	40	Depends on stock solution	Not injected
B	4	10	4000
C	1	4	1000
D	0.25	4	250
E	0.063	4	63
F	0.016	4	16
G	0.0039	4	3.9
H	0.00098	4	1.0

Prepare and calibrate the LC–MS system according to laboratory protocols. Inject and measure the stock solution dilutions from the most dilute to more concentrated, with blank injections between compounds in AIF mode.

Example naming of the files:

InChIKey_stocksolutionID_compound_name_amount(fmol)_date_LCMSmethod_injectionnumber

Example Injection sequence outline:

System preparation and suitability checks. *Blank,* CompoundX_0001fmol, CompoundX_0004fmol, CompoundX_4000fmol, *Blank,* CompoundY_0001fmol,…

Monitor and check the following system parameters during and after acquisition, before proceeding to next step:

- Column pressure and its fluctuations—if out of method specification range and unacceptably fluctuating RT might not be reliable.
- TIC (total ion current)—high background noise and contaminations supress the characterized compound signal and interfere with deconvolution of MS2 spectra from AIF data.
- RT and intensities of the tIS and the characterized compound—RTs and intensities of the tIS should be stable, RT of the characterized compounds stable, and signal increasing with increasing amount injected onto column.

NOTE: (1) Not all compounds are suitable for a given chromatography. For example, an acidic HILIC LC–MS method is suitable for amino acids, nucleosides, and their derivatives, but phosphorylated compounds are poorly detected. Detection of ATP and other nucleotides requires alternative chromatography (e.g., basic HILIC). (2) While some compounds ionize well in positive and negative ionization mode (e.g., amino acids), others are mainly observed in positive (e.g., carnitine and betaine species) or negative (e.g., carboxylic acids). (3) Molecular ion ($[M+H]^+$ or

$[M{-}H]^-$) might not always be the major species. For example, the molecular ions of chenodeoxycholic and cholic acids in positive ionization mode are $[M{+}H{-}2H_2O]^+$ and $[M{+}H{-}3H_2O]^+$. (4) Ion suppression can be caused by co-eluting compounds. In case of HILIC chromatography, sometimes the intensities of late-eluting tIS can be markedly reduced when injecting a concentrated compound (while the intensities of the early eluting tIS remain stable). One possible cause could be ions from the chemical standard (e.g., sodium, chloride) or from solubilization (e.g., dissolving amino acids by addition of HCl to lower the pH).

3. Deconvolution MS2 spectra in MS-DIAL

Step 1. Convert the files from vendor format to mzML

For analysis in MS-DIAL convert the acquired files from vendor format to mzML with *msconvert* program provided in ProteoWizard 3.0 (http://proteowizard.sourceforge.net/) package.
NOTE: (1) Keep all MS levels—no filtering. (2) Files can also be converted using Abf Converter (https://www.reifycs.com/AbfConverter/) to binary abf format supported by MS-DIAL—processing will be faster. However, abf format is currently not supported by many other packages (e.g., XCMS or MZmine) in case troubleshooting is needed or constructing alternative workflow outside of MS-DIAL.

Step 2. Prepare MS-DIAL setting files

Prepare the setting files below to run command line program of MS-DIAL version 3.66 or later.

1. MS-DIAL method file in text format (Figure 2)
2. MS-DIAL DIA setting file in text format (Tab. 2)
3. MS-DIAL tIS file in text format (Tab. 3)
4. Compound library file in MSP format for putative annotation based on MS spectral match only (e.g., all publicly available MS/MS records (drop RTs) from http://prime.psc.riken.jp/Metabolomics_Software/MS-DIAL/). This helps to easier to find the compounds and assess MS spectral matches with other libraries, and assigned InChIKey as well SMILES are useful for later fragment annotation in MS-FINDER.

More detailed descriptions for each of the parameters can be found in the MS-DIAL tutorial (https://mtbinfo-team.github.io/mtbinfo.github.io/MS-DIAL/tutorial#chapter-8).

```
#Data type
MS1 data type: Centroid
MS2 data type: Centroid
Ion mode: Positive
DIA file: F:\CpdChar\_Software_Settings\MS-
DIAL_AIF_project_settings_10_30_0eV.txt

#Data collection parameters
Retention time begin: 0.5
Retention time end: 15
Mass range begin: 40
Mass range end: 1200

#Centroid parameters
MS1 tolerance for centroid: 0.01
MS2 tolerance for centroid: 0.01

#Retention time correction
iSTD file: F:\CpdChar\_Software_Settings\MS-
DIAL_RTcorr_tIS_CpdChar_LC02MS02.txt
Excute RT correction: TRUE
RT correction with smoothing for RT diff: TRUE
User setting intercept: 0
RT diff calc method: SampleMinusReference
Interpolation Method: Linear
Extrapolation method (begin): UserSetting
Extrapolation method (end): LastPoint

#Peak detection parameters
Smoothing method: LinearWeightedMovingAverage
Smoothing level: 3
Minimum peak width: 5
Minimum peak height: 1000
Mass slice width: 0.02

#Deconvolution parameters
Sigma window value: 0.5
Amplitude cut off: 1000
Exclude after precursor: FALSE

#Adduct list
Adduct list: [M+H]+,[M+Na]+,[2M+H]+,[M+H-
H2O]+,[M+H-2H2O]+,[M+K]+

#MSP file and MS/MS identification setting
MSP file: F:\CpdChar\_Software_Settings\MSMS-
Public-Pos-VS12_noRT.msp
Retention time tolerance for identification: 1
Accurate ms1 tolerance for identification: 0.01
Accurate ms2 tolerance for identification: 0.01
Identification score cut off: 60

#Text file and post identification (retention time
and accurate mass based) setting
Text file: F:\CpdChar\_Software_Settings\MS-
DIAL_PostID_tIS_CpdChar_LC02MS02.txt
Retention time tolerance for post identification:
0.5
Accurate ms1 tolerance for post identification:
0.01
Post identification score cut off: 85

#Alignment parameters setting
Retention time tolerance for alignment: 0.1
MS1 tolerance for alignment: 0.015
Retention time factor for alignment: 0.5
MS1 factor for alignment: 0.5
Peak count filter: 20
QC at least filter: FALSE

#CorrDec setting
CorrDec excute: TRUE
CorrDec MS2 tolerance: 0.01
CorrDec minimum MS2 peak height: 500
CorrDec minimum number of detected samples: 4
CorrDec exclude highly correlated spots: 0.9
CorrDec minimum correlation coefficient (MS2): 0.9
CorrDec margin 1 (target precursor): 0.1
CorrDec margin 2 (coeluted precursor): 0.1
CorrDec minimum detected rate: 0.7
CorrDec minimum MS2 relative intensity: 1
CorrDec remove peaks larger than precursor: FALSE
```

Figure 2. MS-DIAL method file contents

Table 2. DIA setting file contents (should be saved as tab separated text file).

ID	MS_Type	Min m/z	Max m/z	Disp_Name	CollisionEnergy	Deconvolution Target
1	ALL	40	1200	10eV	10	1
2	ALL	40	1200	30eV	30	1
0	SCAN	40	1200	0eV	0	1

Table 3. tIS used for RT correction file contents (should be saved as tab separated text file).

Compound/Peak	RT	RT_dev	m/z	Mzdev	intensity	target
Pyrantel STD [M+H]+	2.3	0.5	207.09505	0.01	10000	TRUE
CHES STD [M+H]+	5	0.5	208.10019	0.01	10000	TRUE
5-Fluorocytosine STD [M+H]+	6.1	0.5	130.04410	0.01	10000	TRUE
PIPES STD [M+H]+	9.1	0.8	303.06790	0.01	10000	TRUE
HEPES STD [M+H]+	10.7	0.8	239.10600	0.01	10000	TRUE

<u>Step 3. Prepare MS-DIAL project folder(s)</u>

The command line version of MS-DIAL works on folder basis—all sample files in specified folder will be put into in a single project. Therefore, to characterize compounds one by one, serial dilution files of each compound should be placed into compound-specific directories (Figure 3).

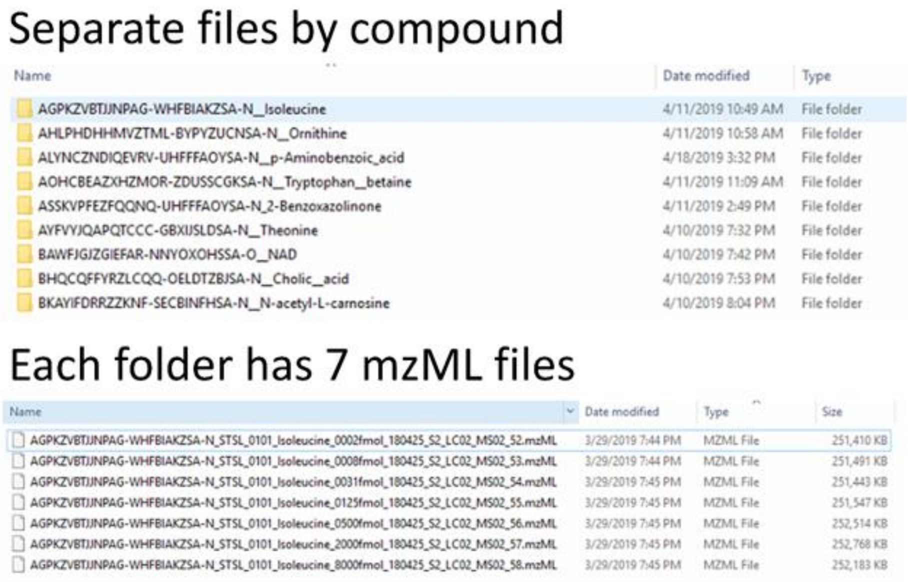

Figure 3. Sort compound files into separate folders

Step 4. Run MS-DIAL console program

Run the following command on Command Prompt after changing user specific settings in **bold font**. One command works for one compound per project (Figure 4). The benefit of using command line is scaling (many compounds can be automatically processed in a queue using .bat file containing commands for multiple compounds) and relatively short processing time—not the whole m/z and RT range is processed, but only a specified target m/z.

path_to_MsdialConsoleApp.exe lcmsdia -p -mCE -i **input_dir** -o **output_dir** -m **path_to_method_file** -target **target_m/z**

A specific example for trigoneline below, full command in Figure 4:

path_to_MsdialConsoleApp.exe:
F:\CpdChar_Software_Settings\MSDIAL_v3.98\MsdialConsoleApp.exe
input_dir:
F:\CpdChar\WWNNZCOKKKDOPX-UHFFFAOYSA-N__Trigonelline
output_dir:
F:\CpdChar\WWNNZCOKKKDOPX-UHFFFAOYSA-N__Trigonelline
path_to_method_file:
F:\CpdChar_Software_Settings\MS-DIAL_LCMS-AIF-CmdLine_Parameters.txt
target_m/z:
138.05495

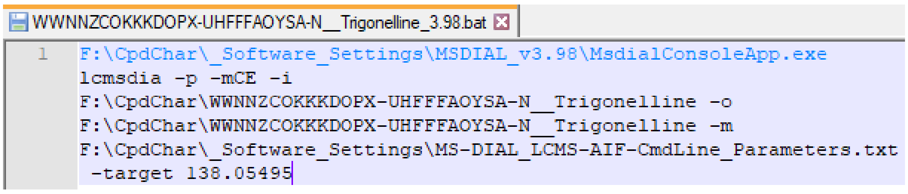

Figure 4. Screenshot of MS-DIAL command for console (if saved in a single text line and file ending is .bat, the command can be easily executed by a double click).

MS-DIAL console version during data processing will generate a report on RT corrections (Figure 5).

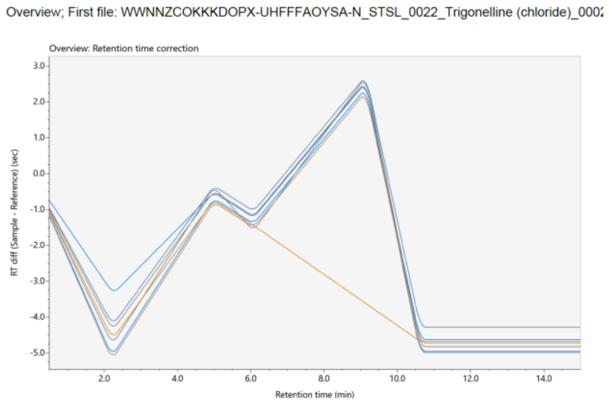

Figure 5. Example RT correction report for trigonelline generated by MS-DIAL console version.

Step 5. Inspect the deconvoluted MS spectra in MS-DIAL and export to MS-FINDER.

After successful processing by the MS-DIAL console version, the project directory contains all the necessary files to be opened by a regular MS-DIAL version. Using the graphical user interface, various parameters can be inspected and adjusted as shown in Figure 6 (e.g., RTs, peak shapes and heights, etc). Then, deconvoluted MS spectra are exported to MS-FINDER, as shown in Figure 7 (MS2Dec) and Figure 8 (CorrDec).

High-quality MS spectra are preferable for library construction. While MS spectra resulting from low-intensity signal tend to be poor and noisy, MS spectra from saturated signals may contain fragments usually not observed in the samples, and mass accuracy can be affected. Therefore, when selecting MS2Dec deconvoluted spectra for the library, choose a file with high abundance, but not saturated signal. In the case of trigonelline (Figure 6), the three injections with highest concentration (pink, green, brown) are high-abundant or saturated (>1E6). Therefore, to obtain optimal MS2Dec deconvoluted spectra, the fourth most concentrated injection was chosen (Figure 7). MS2Dec MS spectra from saturated and low-abundant files can also be exported for reference. MS spectra exported in MSP format will contain the file name from which the MS spectra was deconvoluted in the comment.

CorrDec deconvolutes the MS spectra based on the precursor–fragment correlations between the samples, and works most efficiently in large and variable sample sets. However, when only few files are available, as in case of the compound characterization, a highly saturated precursor signal can affect the deconvoluted MS spectra. More specifically, saturated precursor signal produces a reduced correlation with the fragment signal, which might still be increasing. Therefore, inspection is necessary, and in some cases, parameters need adjustment (Figure 8). As a rule of thumb, the MS2Dec and CorrDec spectra should be similar in the case of pure chemical standard characterization.

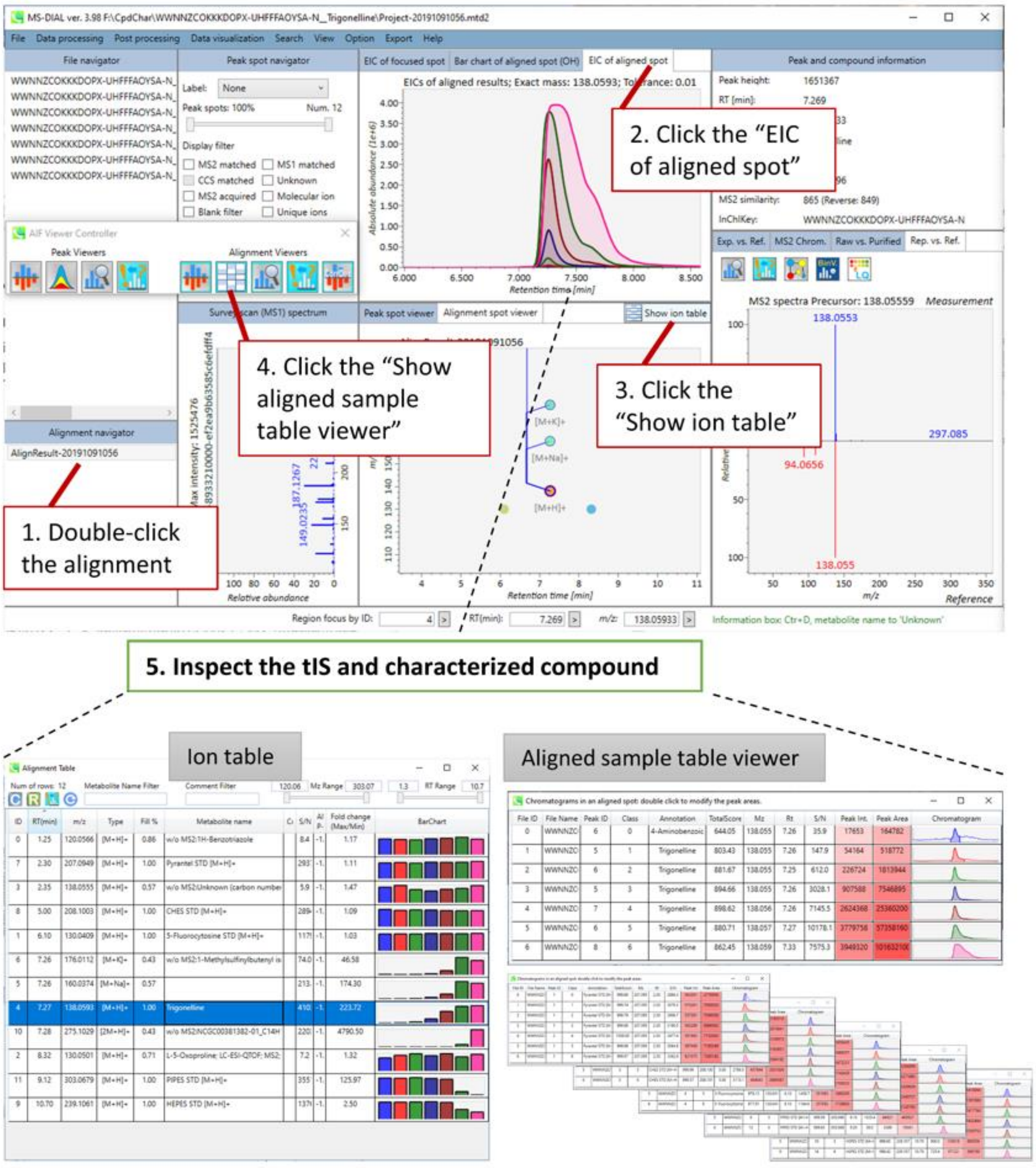

Figure 6. Inspection of the compound characterization project created by the MS-DIAL console version.

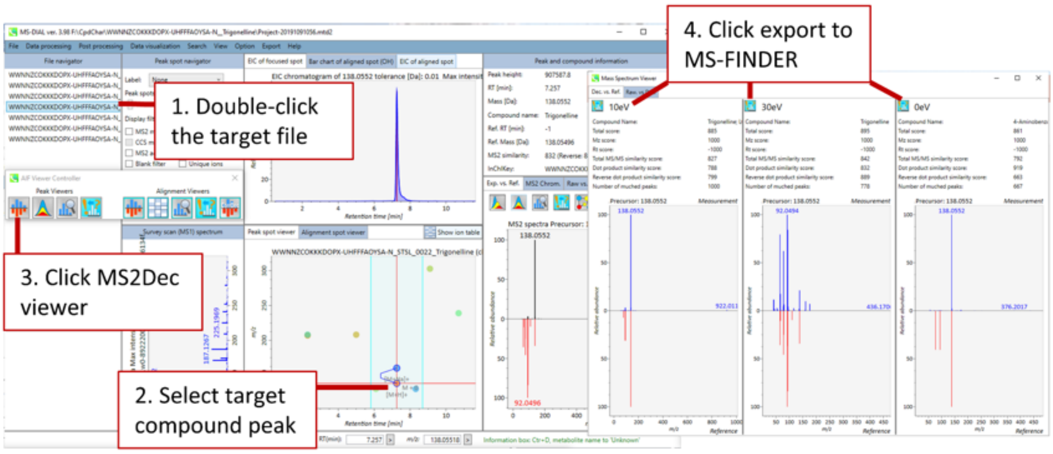

Figure 7. Export MS2Dec spectra from MS-DIAL to MS-FINDER

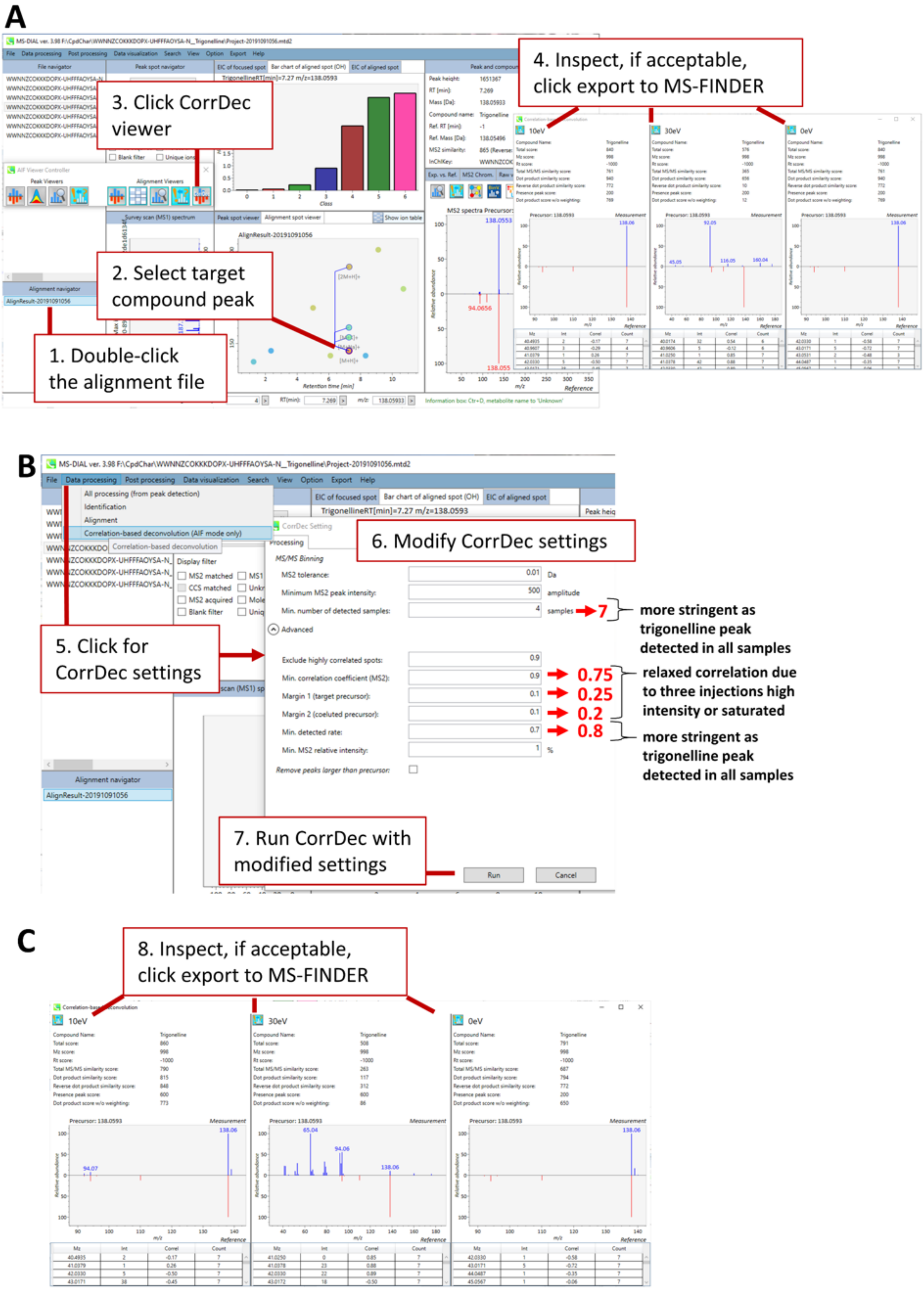

Figure 8. Inspecting CorrDec MS spectra of trigonelline (**A**), adjusting parameters and re-running CorrDec (**B**), exporting to MS-FINDER (**C**).

4. Annotation of MS fragments in MS-FINDER

MS-FINDER version 3.22 or later is required for annotation of deconvoluted MS spectra from AIF data. MS-FINDER settings need to be changed as shown in Figure 9 to annotate adducts above the precursor m/z.

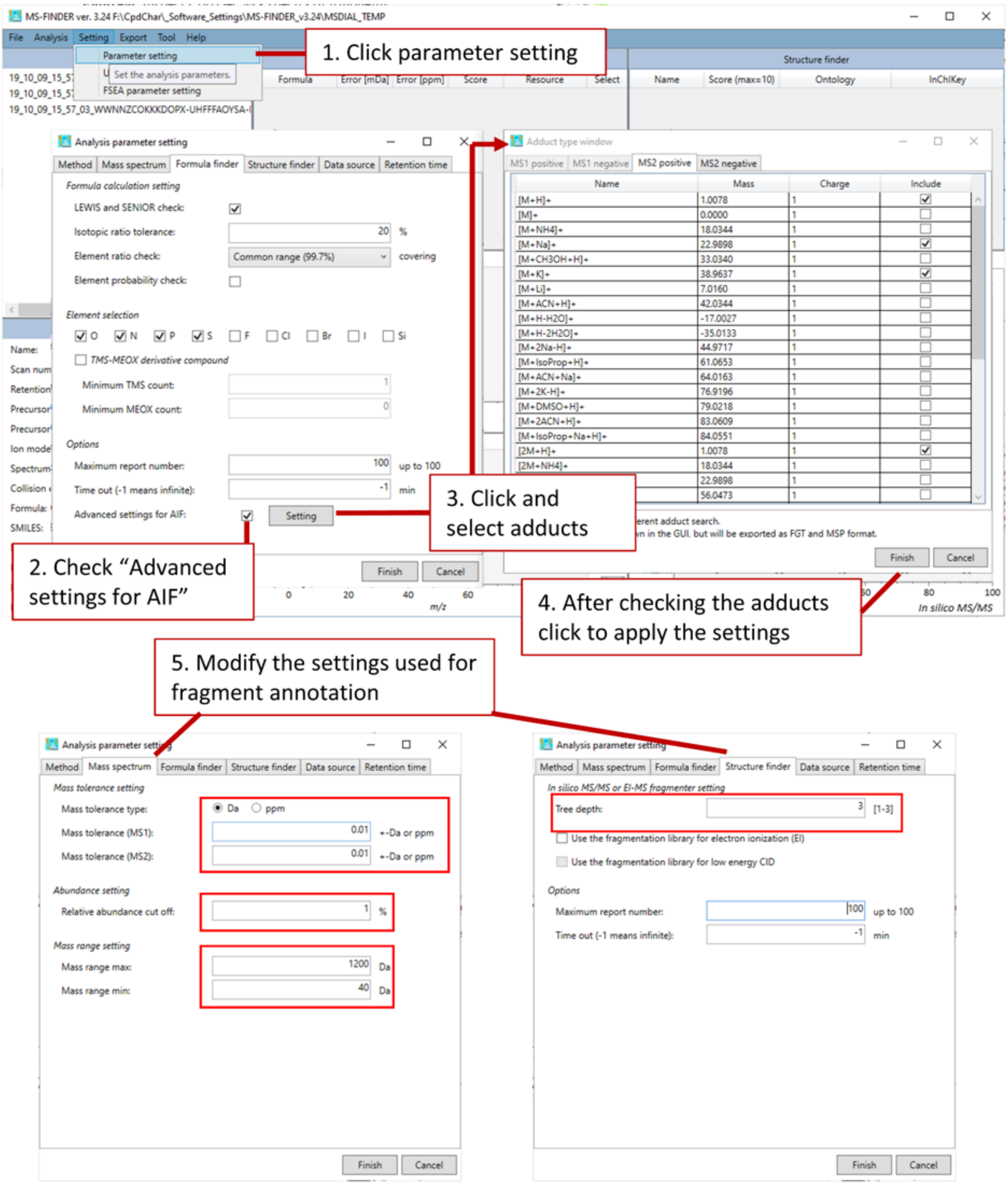

Figure 9. MS-FINDER settings to annotate fragments above the precursor mass in positive ionization mode.

MS spectra exported from MS-DIAL (Figure 7 and 8) to MS-FINDER are added to the bottom of the list in the file navigator, with a time stamp in front (Figure 10A). If a library was already used in MS-DIAL for identification (e.g., MS2 DIA library without retention times), then the file information may already be present (Figure 10A). Compound information also can be obtained in MS-FINDER

by using the Molecular Formula Finder (double click a spectrum in the file navigator) and then Structure Finder (right click in the Molecular Formula Finder), which should result in the characterized compound being among the top hits (matching InChIKey with chemical standard). File information for each spectrum can be added by copy–pasting from the Structure Finder Meta data panel (Figure 10A). Of particular importance are the molecular formula and SMILES, as MS-FINDER will use this information to annotate the fragments. Then, run fragment annotation as batch job (Figure 10B), and export the result as MSP format (Figure 10C).

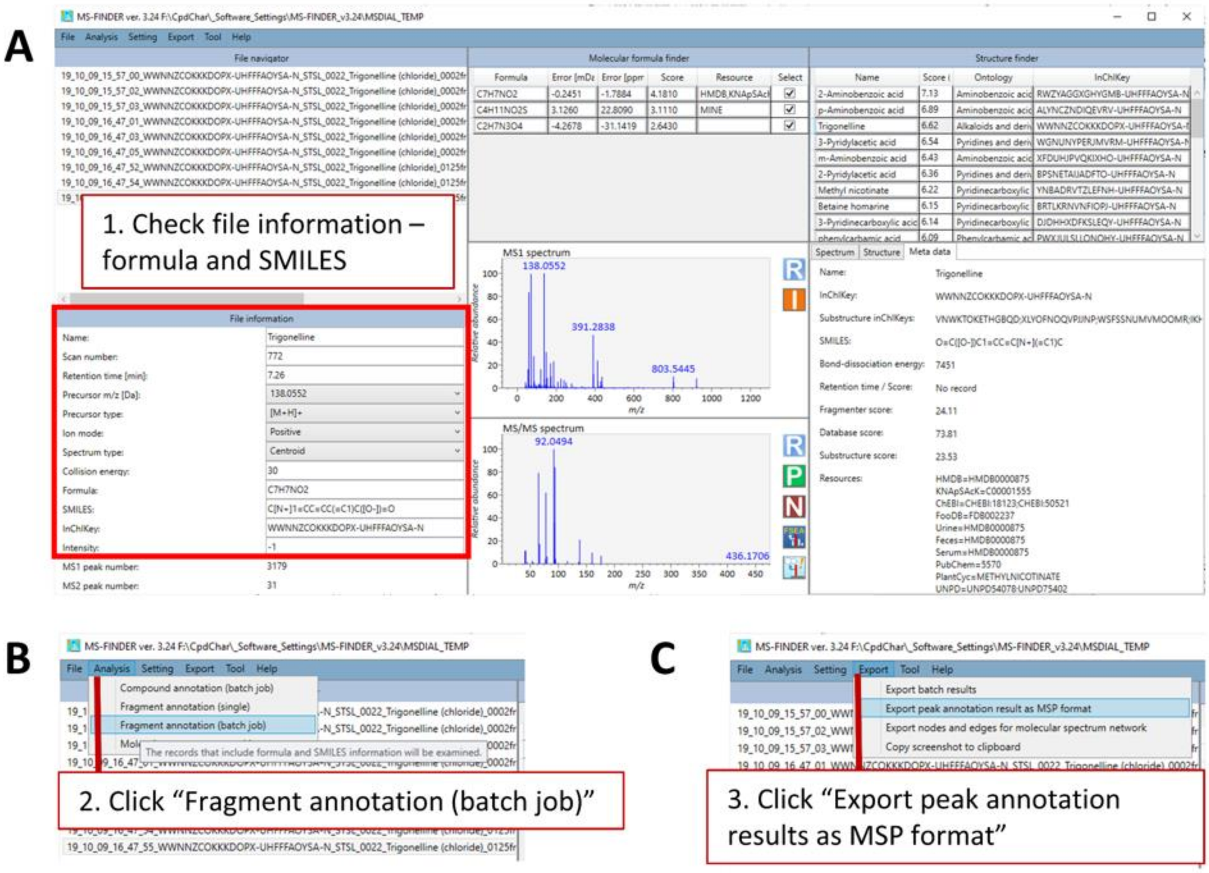

Figure 10. Inspection of the imported MS spectra information (A), fragment annotation (B), and export to MSP file (C).

5. Library assembly and curation in MS-LIMA

MSP files exported by MS-FINDER are then imported into MS-LIMA (Figure 11A). When handling multiple compound-specific MSP files at the time, they can be combined into a single MSP file using a text editor. During the import of MSP file containing multiple spectral records into MS-LIMA, a couple of sanity checks will be performed (do all the records with the same InChIKey have the same chemical formula? Do spectral records with the same InChIKey have similar RT?) and warnings issued. Settings for the checks during the import and MS spectrum visualizations can be modified in the settings window (Figure 11A).

After importing the MSP file, MS spectral records grouped by compound (using InChIKey) are viewed (Figure 11B) and their quality assessed (e.g., mass accuracy of precursor ions, peak annotations etc.). Among multiple options to view the MS spectra, MS-LIMA also contains a comparative spectral viewer (Figure 11C) for MS spectra from either the same or different MSP files. MS-LIMA can export MS spectra visualizations and comparisons as publication-quality PNG or EMF files.

Mass editing of the records, such as removal of unannotated peaks, conversion of precursor *m/z* to theoretical *m/z*, and updating common metadata (method, instrument type etc) is available in the utility menu (Figure 11A). More complex and specific modifications of the MSP files not covered by MS-LIMA can be performed using a text editor.

NOTE: In MSP format, there are unfortunately no strict rules for describing meta information. For example, "retention_time" and "RETENTIONTIME" can be used to indicate RT in MS-LIMA. Metadata lines can be included for each record in AMRT+MS2 library (e.g., full InChI, experimental conditions etc.) without affecting MS-LIMA functionality.

For the AIF spectra, we suggest to inspect and curate the MS spectra using MS-LIMA as below:

- Convert precursor *m/z* to theoretical *m/z*—it is known which chemical standard is characterized. The mass accuracy of the MS spectra can be assessed in the "peaks and annotation" panel Figure 11B.
- Remove the nonannotated peaks by MS-FINDER—reduce noise
- Cut off peaks with <1% intensity—settings in MS-FINDER (Figure 9)—reduce noise
- Inspect the MS spectra and remove peaks with low mass accuracy (e.g., >20ppm, while the others are <5ppm)—might be miss annotation or noise peak.
- Check and add metadata (e.g., full InChI, experimental conditions)

NOTE: All the curations and editing of the MS spectral records will not affect original MSP file. To save the modifications, the edited MSP file has to be exported to a new MSP file. Autobackup option is also available (file will be autoexported at intervals specified in the settings). Detailed explanation of MS-LIMA software is available (https://github.com/tipputa/MS-LIMA-Standard).

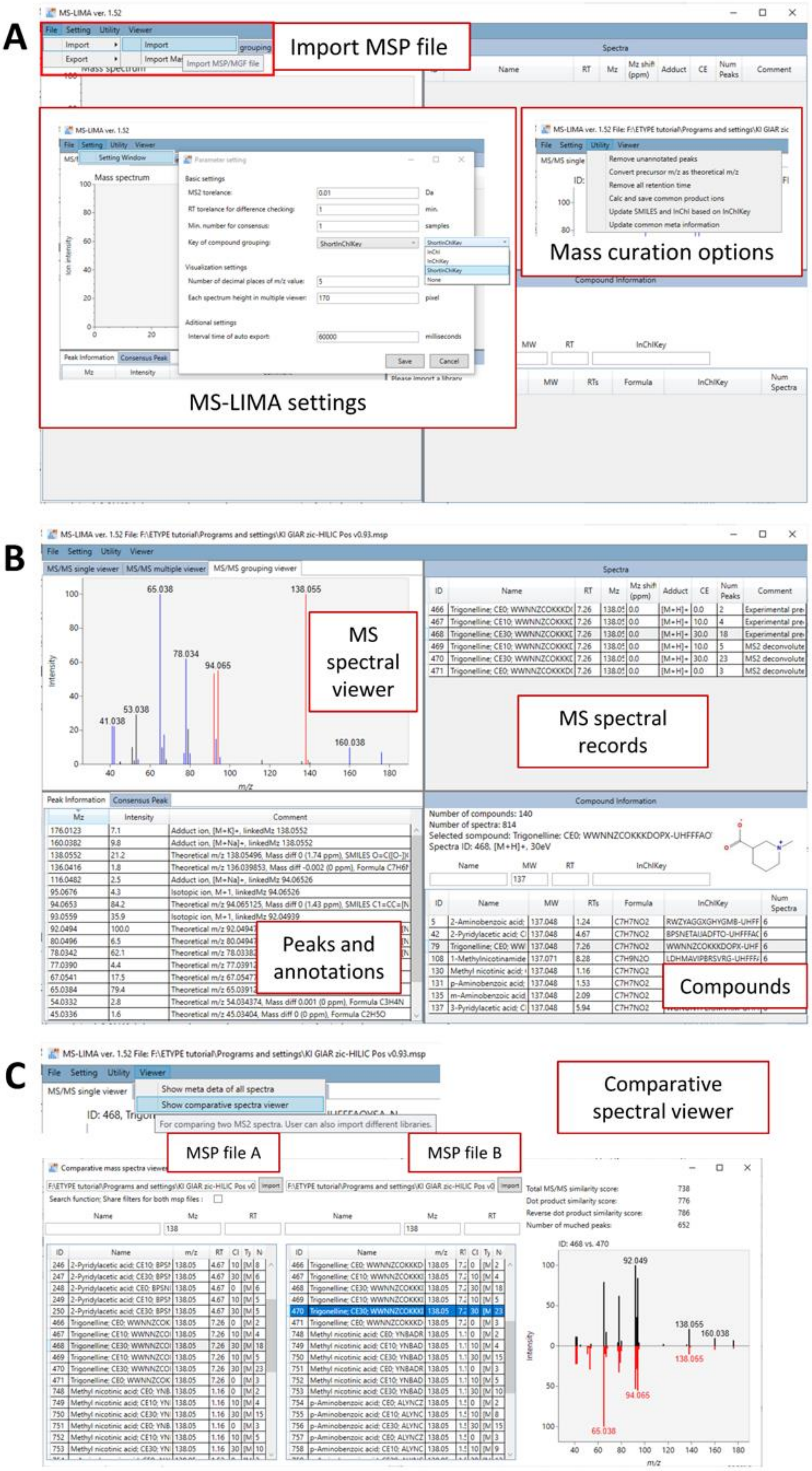

Figure 13. MS-LIMA settings and tools (A), main window after MSP library import (B), and comparative MS spectral viewer (C).
