# Supplementary material for: Creating a Reliable Mass Spectral–Retention Time Library for All Ion Fragmentation-Based Metabolomics
Source: Metabolites. 2019 Oct 26;9(11):251. doi: 10.3390/metabo9110251 (PMC6918128; doi:10.3390/metabo9110251)
Supplement: Supplementary file 1 [file metabolites-09-00251-s001.zip › supplementary materials/Tutorial 2. Compound identification in LC-MS AIF data using AMRT MS2 library.pdf]

Identification of compounds in a metabolomics dataset acquired in AIF mode using LC–MS-method-specific AMRT+MS2 library in MS-DIAL

Content

1. OVERVIEW 2

2. DATASET, SOFTWARE AND SETTING FILES 2

3. PROCESSING DATASET ACQUIRED USING AIF MODE 4

4. REVIEWING COMPOUND ANNOTATIONS 11

5. NOTES 19

If used in data processing for publication, please cite:
Tada, I.; Tsugawa, H.; Meister, I.; Zhang, P.; Shu, R.; Katsumi, R.; Wheelock, C.E.; Arita, M., Chaleckis R. Creating a Reliable Mass Spectral–Retention Time Library for All Ion Fragmentation-Based Metabolomics. *Metabolites* **2019**

Contact: Romanas Chaleckis (romcha@gunma-u.ac.jp), International Open Laboratory (Karolinska Institutet) at Gunma Initiative for Advanced Research (GIAR), Japan.

1. Overview

Here we provide a short step-by-step MS-DIAL tutorial for processing all ion fragmentation (AIF) data, focusing on the compound identification using accurate mass, retention time, and MS spectral library (AMRT+MS2). A flowchart of the workflow is shown in Figure 1.

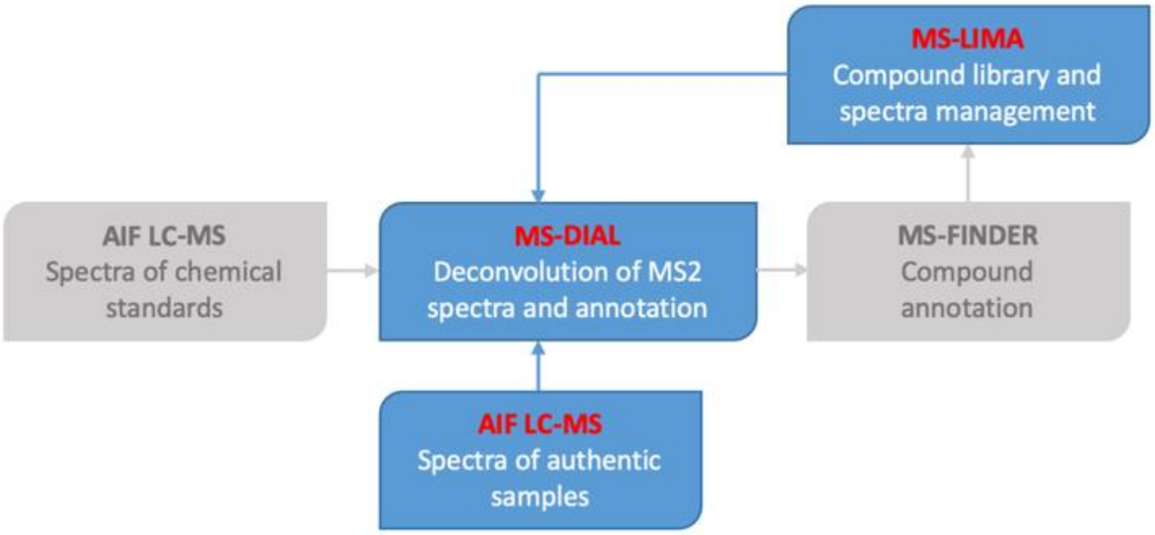

Figure 1. Flowchart of the workflow for annotating AIF metabolomics dataset using method-specific AMRT+MS2 library

More detailed and in-depth information on how to use MS-DIAL and related software is available on RIKEN PRIME webpage (http://prime.psc.riken.jp/Metabolomics_Software/MS-DIAL/), detailed tutorial for MS-DIAL is also available (https://mtbinfo-team.github.io/mtbinfo.github.io/MS-DIAL/tutorial).

2. Dataset, software, and setting files

Metabolomics dataset acquired in AIF mode

Metabolights MTBLS816: https://www.ebi.ac.uk/metabolights/MTBLS816

Samples: Urine samples (n = 224, from an asthma study (PMID: 29518425) and quality control (QC) sample pooled from all study samples were first normalized to a same specific gravity value of 1.0025 by the addition of water. Then, samples were diluted 10-fold with acetonitrile containing technical internal standards (tIS), see Step 6 in the data processing section. Samples were measured in four analytical batches, each batch consisting of 49–63 samples, with QC sample injections every five samples, and a water blank at the end of the batch sequence.

Data acquisition: LC–MS measurements in AIF mode were performed as described previously (PMID: 28641411, PMID: 29363064). Briefly, metabolite separation was performed on Agilent 1290 Infinity II system using HILIC SeQuant ZIC-HILIC (Merck, Darmstadt, Germany) column 100 Å (100 × 2.1 mm, 3.5 μm particle size) coupled to a guard column (2.1 × 2 mm, 3.5 μm particle size). Sample analysis was performed using water with 0.1% formic acid (pH 2.63; solvent A) and acetonitrile with 0.1% formic acid (solvent B). The elution gradient used was as follows: isocratic step at 95% B for 1.5 min, 95% to 40% B in 12 min, maintained at 40% B for 2 min, then decreasing to 25% B at 14.2 min, maintained for 2.8 min, then returned to initial conditions over 1 min, and a column re-equilibration phase of 7 min. The flow rate was 0.3 mL/min, the injection volume 2 μL, and the column oven temperature 25 °C. Data was acquired in positive ionization mode on an Agilent 6550 Q-TOF-MS system in centroid mode. Nitrogen was used as sheath gas and drying gas at a flow of 8 and 15 L/min, respectively. The drying and sheath gas temperature was set at 250 °C,

with the nebulizer pressure at 35 psig, and voltage 3000 V. Data was obtained with a mass range of 40−1200 *m/z* in AIF mode, including three sequential experiments at alternating collision energies: one full scan at 0 eV, followed by one MS/ MS scan at 10 eV, and then followed by one MS/MS scan at 30 eV. The data acquisition rate was 6 scans/s.

Software

- Abf Converter: https://www.reifycs.com/AbfConverter/_
  conversion of the LC–MS vendor file formats into binary Abf format used by MS-DIAL.
- (optional) ProteoWizard: http://proteowizard.sourceforge.net/_
  conversion and cropping of the various LC-MS file formats
- MS-DIAL: http://prime.psc.riken.jp/Metabolomics_Software/MS-DIAL/
  Universal program for metabolomics data processing. Version 3.22 and above have two methods for deconvoluting MS spectra from AIF data: MS2Dec (original MS-DIAL method, works on single files/samples) and CorrDec (publication in preparation, requires multiple files for MS spectra deconvolution, applicable for multisample/cohort studies).

Setting files

All the settings files (except the AMRT+MS2 library) can be downloaded as a zipped file from the RIKEN PRIMe website (http://prime.psc.riken.jp/Metabolomics_Software/MS-DIAL/aiftutorial.zip or http://prime.psc.riken.jp/)

- AIF experiment file
  A tab separated text file describing the MS spectra contained in the LC–MS files (see Step 3 in the data processing section).
- (Optional) sample information
  A spreadsheet with the information on the dataset samples (see Step 4 in the data processing section). Not directly needed for compound identification using AMRT+MS2 library, but specifying at least sample type (QC, sample or blank) can provide useful information on the compound to annotate. Values can be entered manually, however using a spreadsheet is time-saving when processing hundreds of samples.
- (Optional) MS-DIAL settings
  A single file containing all the data processing settings, except the RT correction (Step 5 in the data processing section). Settings files should be used with the same version of the MS-DIAL in which it was created (otherwise MS-DIAL may crash, file following the link above is for MS-DIAL version 3.98). Alternatively, the parameter values can be entered manually.
- (Optional) peaks for RT correction
  A spreadsheet or tab separated text file containing the peaks to be used for RT drift assessment and corrections (see Step 6 in the data processing section). Alternatively, the parameter values can be entered manually.
- AMRT+MS2 library file
  (http://prime.psc.riken.jp/Metabolomics_Software/MS-DIAL/KI-GIAR_zic-HILIC_Pos_v0.90.msp)
  MSP file containing 140 compounds (814 spectra) with RT for LC–MS AIF method used for the urinary metabolomics dataset acquisition. See publication for details on the creation of the library for AIF data.

3. <u>Processing dataset (AIF mode)</u>

• <u>Step 1. File conversion</u>

Data files should be converted to Abf format using Abf Converter (Figure 2) for processing in MS-DIAL.

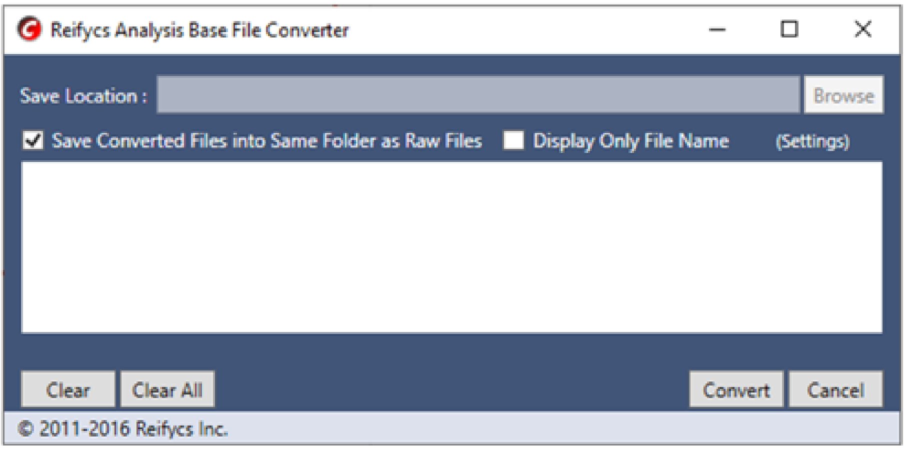

Figure 2. Drag and drop the files into Abf Converter window

<u>NOTE:</u> (1) MS-DIAL can also use mzML format, however data processing will be faster using Abf files (binary format—smaller files, faster access), (2) if cropping of the files is needed before conversion to Abf format, *msconvert* program provided in the ProteoWizard package can be used (keep all MS levels—no filtering).

• <u>Step 2. Prepare the files and information needed for the MS-DIAL project.</u>

Copy all Abf files into a study folder (files created by MS-DIAL will be later placed in the same folder). Furthermore, you will need AIF experimental file, (optional) sample information file, (optional) MS-DIAL settings file, peaks for RT correction information (optional), and AMRT+MS2 library file.

<u>NOTE:</u> We recommend to create inside the study another folder where the MS-DIAL program version used for creating the project as well as setting files are stored (Figure 3).

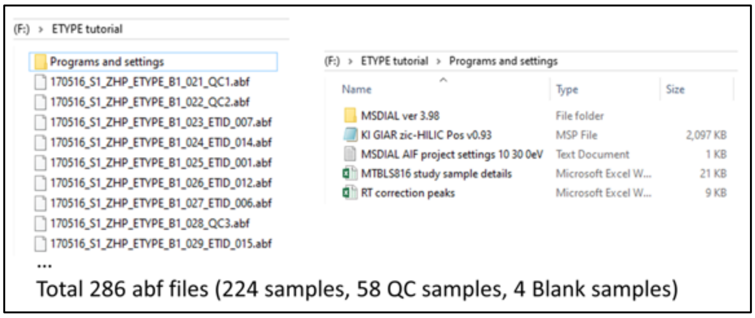

Figure 3. Prepared files and folders for MS-DIAL project

• <u>Step 3. Start MS-DIAL project.</u>

Set the parameters in the initial MS-DIAL start up window as described in Figure 4 and press “Next”.

A

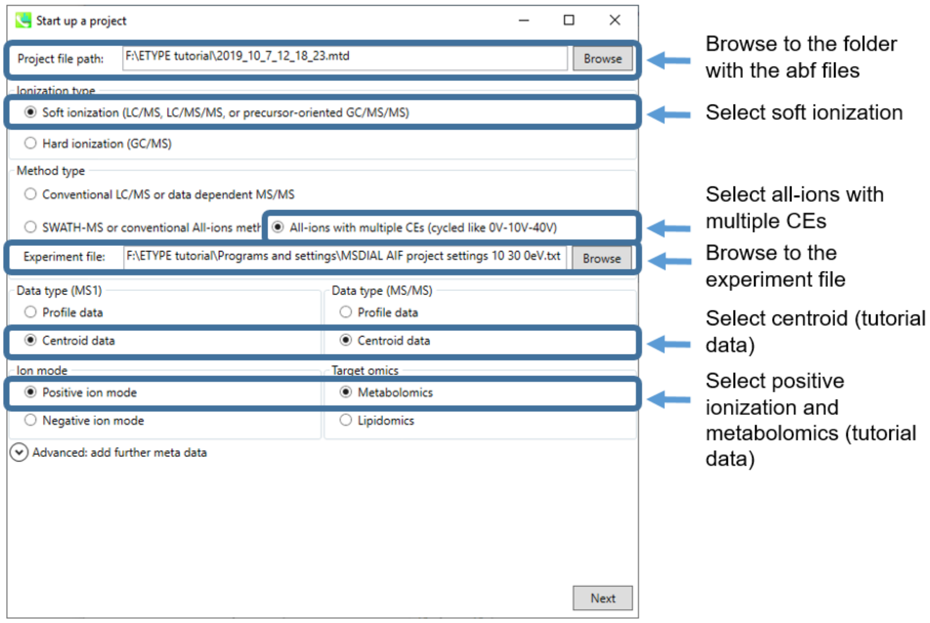

B

Experiment	MS Type	Min m/z	Max m/z	Display Name	Collision Energy	Deconvolution Target
1	ALL	40	1200	10eV	10	1
2	ALL	40	1200	30eV	30	1
0	SCAN	40	1200	0eV	0	1

Figure 4. Starting MS-DIAL AIF project. (A) Initial settings window and (B) experiment file contents for the tutorial data (formatted as tab-delimited text file). In the "experiment" column, the experiment ID is listed (by order of acquisition, usually vendor-specific), "MS Type" defines whether the spectrum is all ion fragmentation (ALL) or MS1 (SCAN), "Min m/z" and "Max m/z" define the *m/z* range of the data to be used in the project, "Display Name" and "Collision energy" provide the description of the spectra in MS-DIAL and when exporting as MSP file (e.g., for annotation in MS-FINDER), "Deconvolution target" if set to "1" will perform the MS2Dec deconvolution on the experiment (if set "0", no MS2Dec deconvolution will be performed).

• <u>Step 4. Select Abf files and input sample information.</u>

After setting the initial parameters, select the Abf files for processing, add sample information to the MS-DIAL as shown in Figure 5, and press "Next".

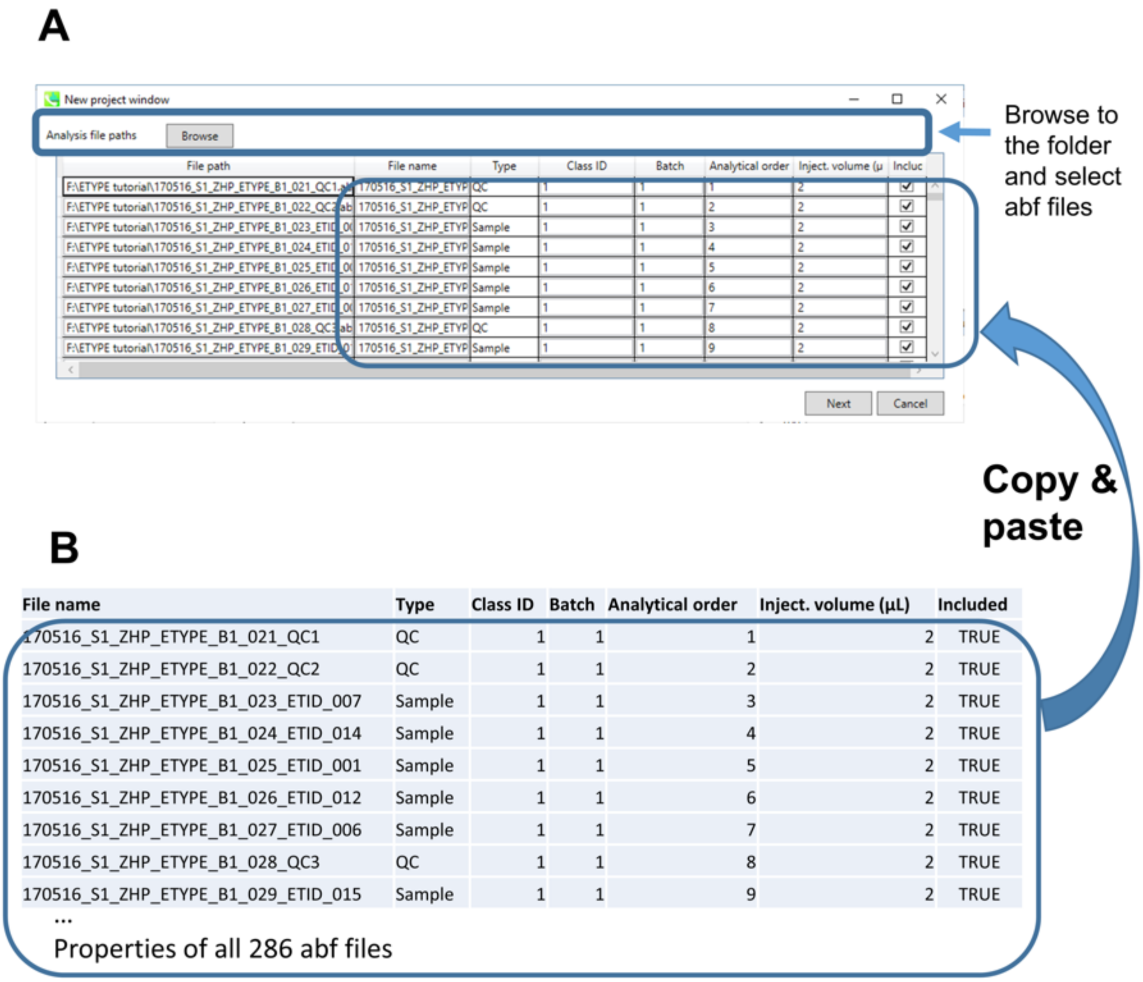

File name	Type	Class ID	Batch	Analytical order	Inject. volume (μL)	Included
170516_S1_ZHP_ETYPE_B1_021_QC1	QC	1	1	1	2	TRUE
170516_S1_ZHP_ETYPE_B1_022_QC2	QC	1	1	2	2	TRUE
170516_S1_ZHP_ETYPE_B1_023_ETID_007	Sample	1	1	3	2	TRUE
170516_S1_ZHP_ETYPE_B1_024_ETID_014	Sample	1	1	4	2	TRUE
170516_S1_ZHP_ETYPE_B1_025_ETID_001	Sample	1	1	5	2	TRUE
170516_S1_ZHP_ETYPE_B1_026_ETID_012	Sample	1	1	6	2	TRUE
170516_S1_ZHP_ETYPE_B1_027_ETID_006	Sample	1	1	7	2	TRUE
170516_S1_ZHP_ETYPE_B1_028_QC3	QC	1	1	8	2	TRUE
170516_S1_ZHP_ETYPE_B1_029_ETID_015	Sample	1	1	9	2	TRUE

Figure 5. Selecting the files for MS-DIAL project. (A) Selecting the files and (B) copying the information of the hundreds of samples from a prepared spreadsheet (simple copy paste is possible into some of the MS-DIAL dialog windows).

• <u>Step 5. Parameter settings in MS-DIAL</u>

A file containing parameter settings can be loaded from a previously saved file by clicking "Load" (Figure 6–9). Parameter values for the tutorial data are shown with brief comments in Figures 6 to 9. More detailed descriptions for each of the parameters can be found in the MS-DIAL tutorial (https://mtbinfo-team.github.io/mtbinfo.github.io/MS-DIAL/tutorial).

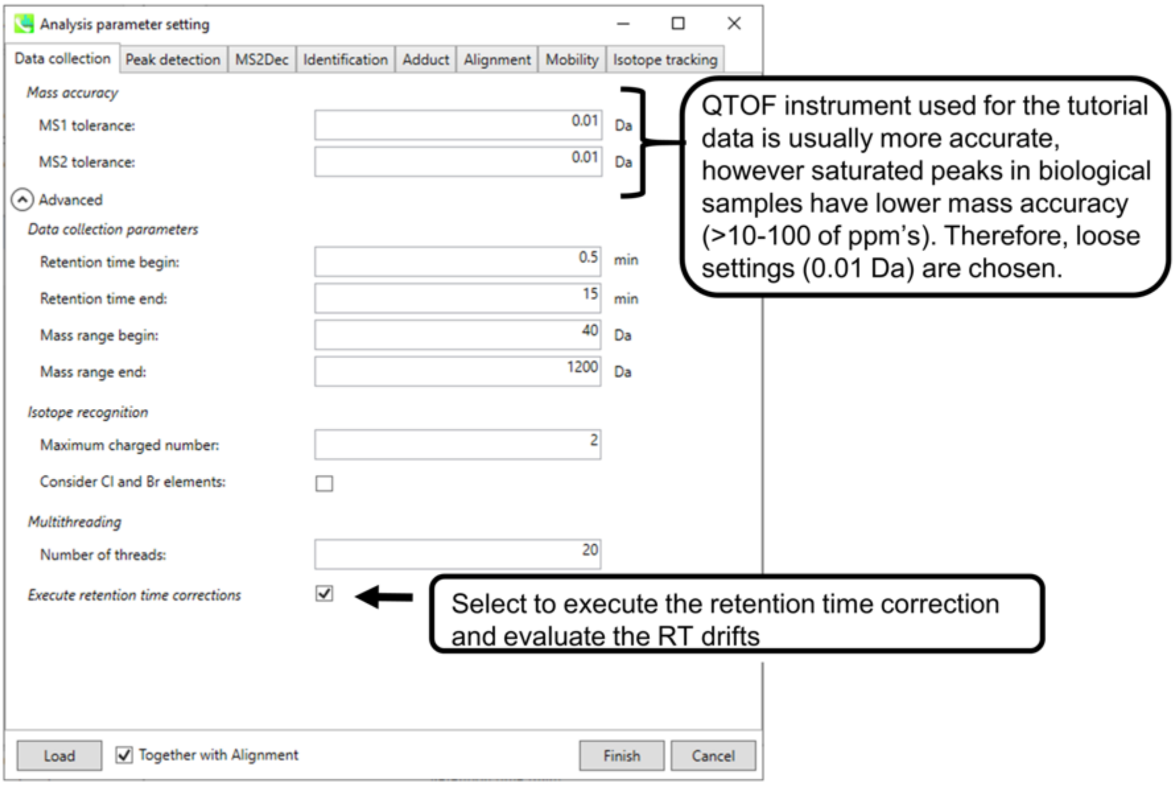

Figure 6. Settings for the Data Collection tab of the MS-DIAL project.

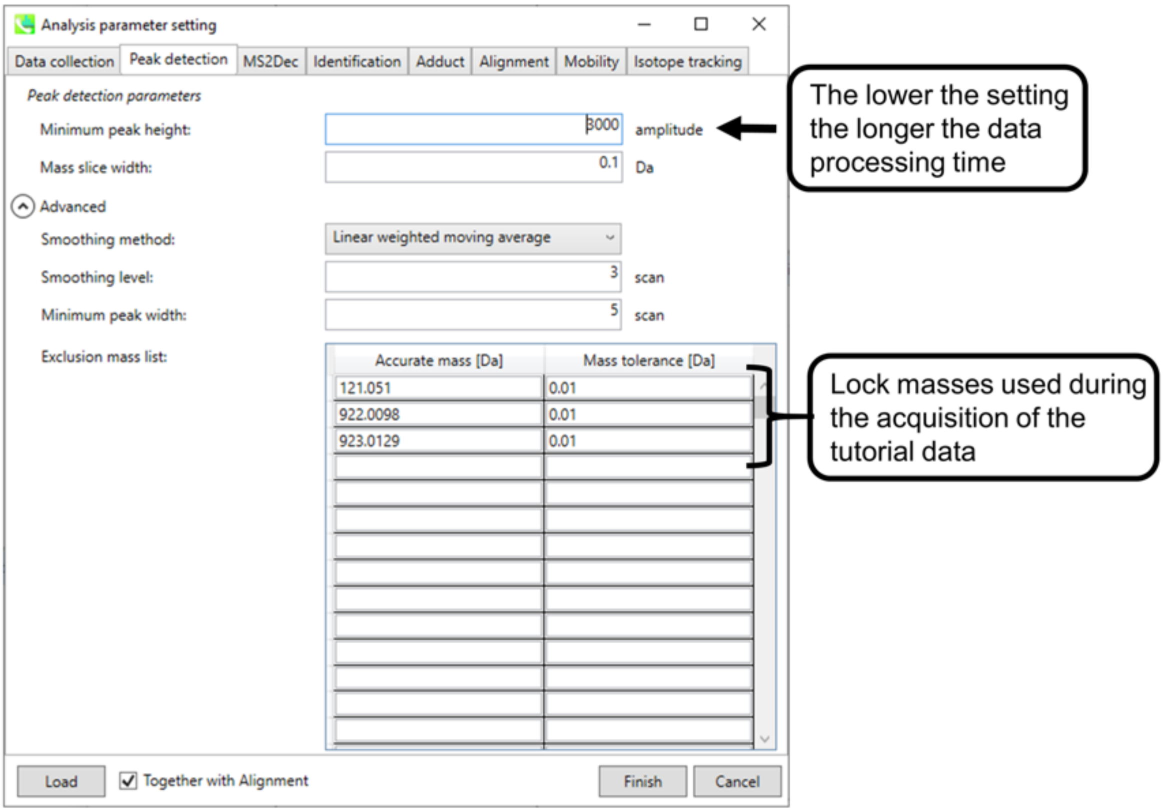

Figure 7. Settings for the Peak Detection tab of the MS-DIAL project.

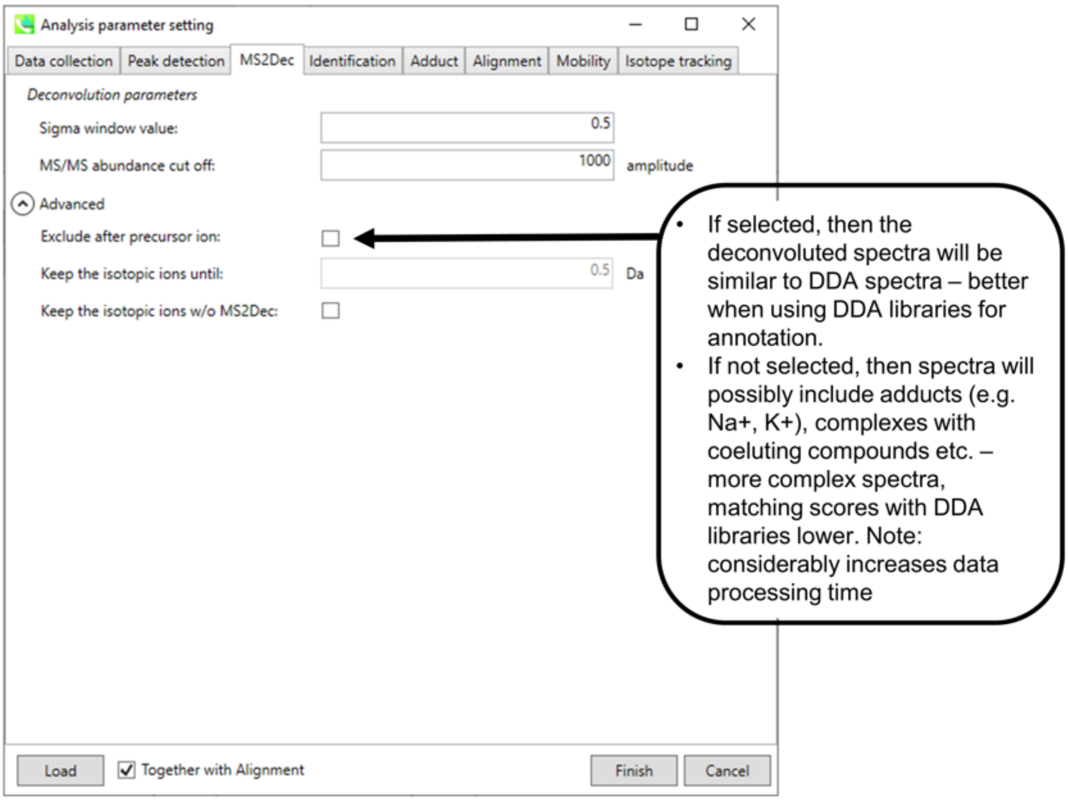

Figure 8. Settings for the MS2Dec tab of the MS-DIAL project.

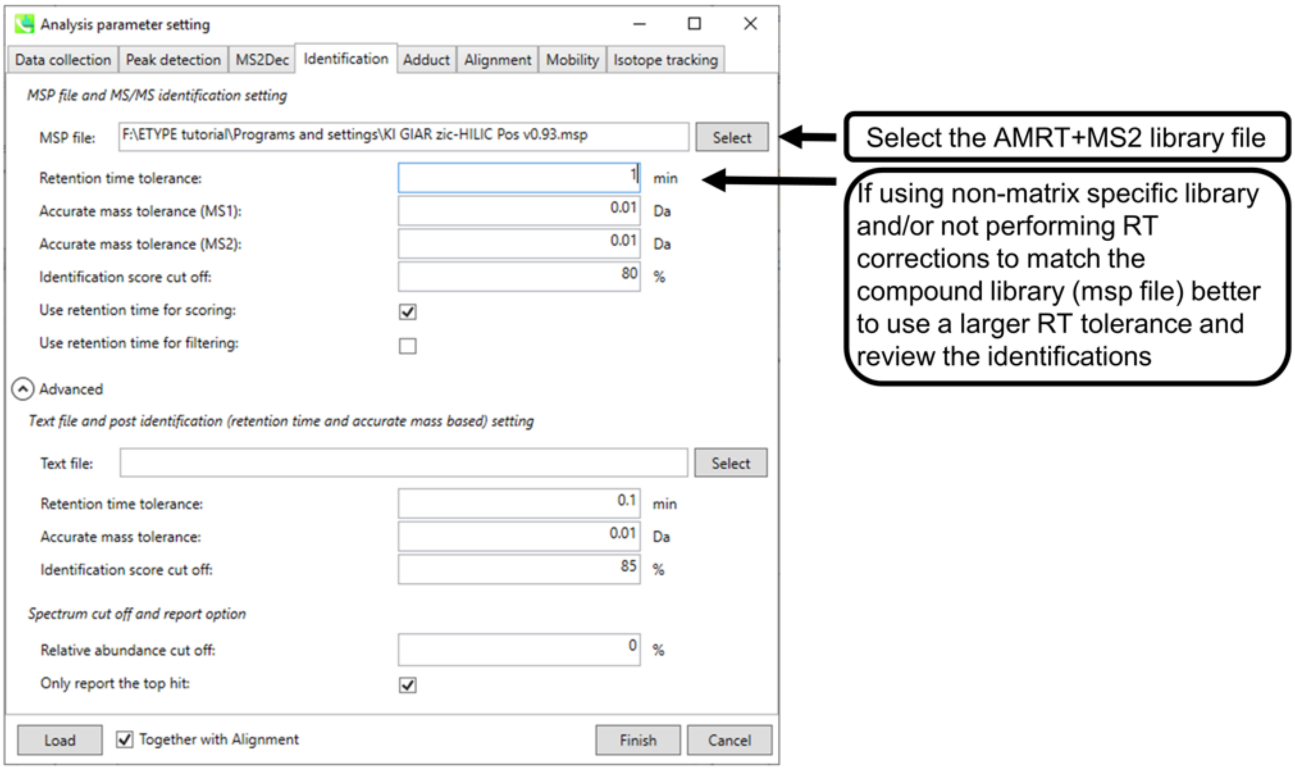

Figure 9. Settings for the Identification tab of the MS-DIAL project.

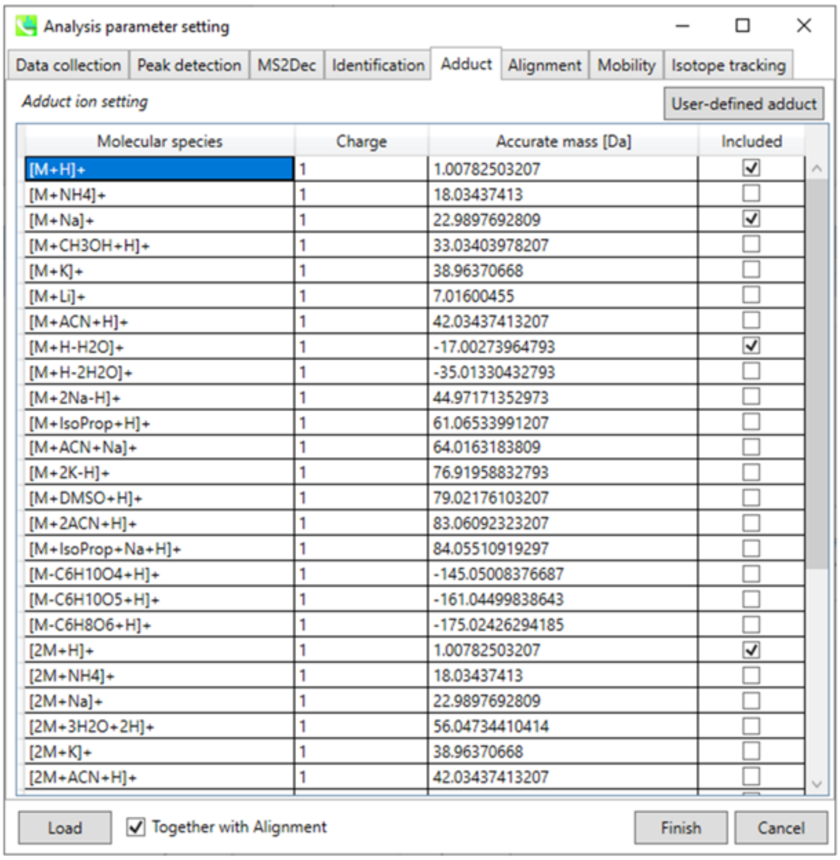

Figure 10. Settings for the Adduct tab of the MS-DIAL project.

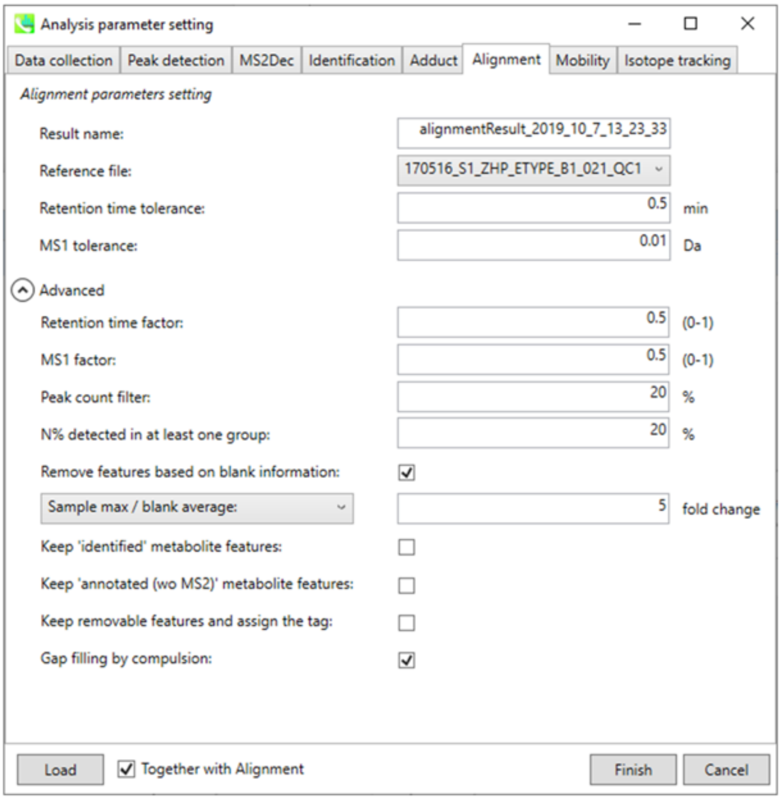

Figure 11. Settings for the Alignment tab of the MS-DIAL project.

Mobility and Isotope tracking tabs are not used in the tutorial. After setting the parameters, click “Finish” to proceed to the retention time correction (if selected, Figure 6) or start data processing straight away.

• <u>Step 6. Retention time correction.</u>

In the first step of data processing, the retention time correction window will open to set the reference compounds (Figure 12) to correct for RT drifts between the cohort samples and also try to match the RT with the AMRT+MS2 library. In the dataset used for the present tutorial, four technical internal standards (tIS) were used: CHES, MES, PIPES, and HEPES, and only three of them (CHES, PIPES, and HEPES) overlap with the tIS used for the construction of the AMRT+MS2 library used for annotation (please note that the tutorial dataset was acquired during the early phase of tIS development, hence the discrepancy).

As shown in Figure 12, for the calculation of the RT difference, select “Sample-Reference”, paste the information and select the peaks to be used for the RT correction, and press “RT correction” button. Usually after a minute(s), an RT correction overview will appear. In the case of the tutorial data, the deviations are <0.5 min. The RT tolerance of 1 min for the identification of the AMRT+MS2 library (Figure 9) is reasonable as it also takes into consideration possible RT shifts due to matrix effects.

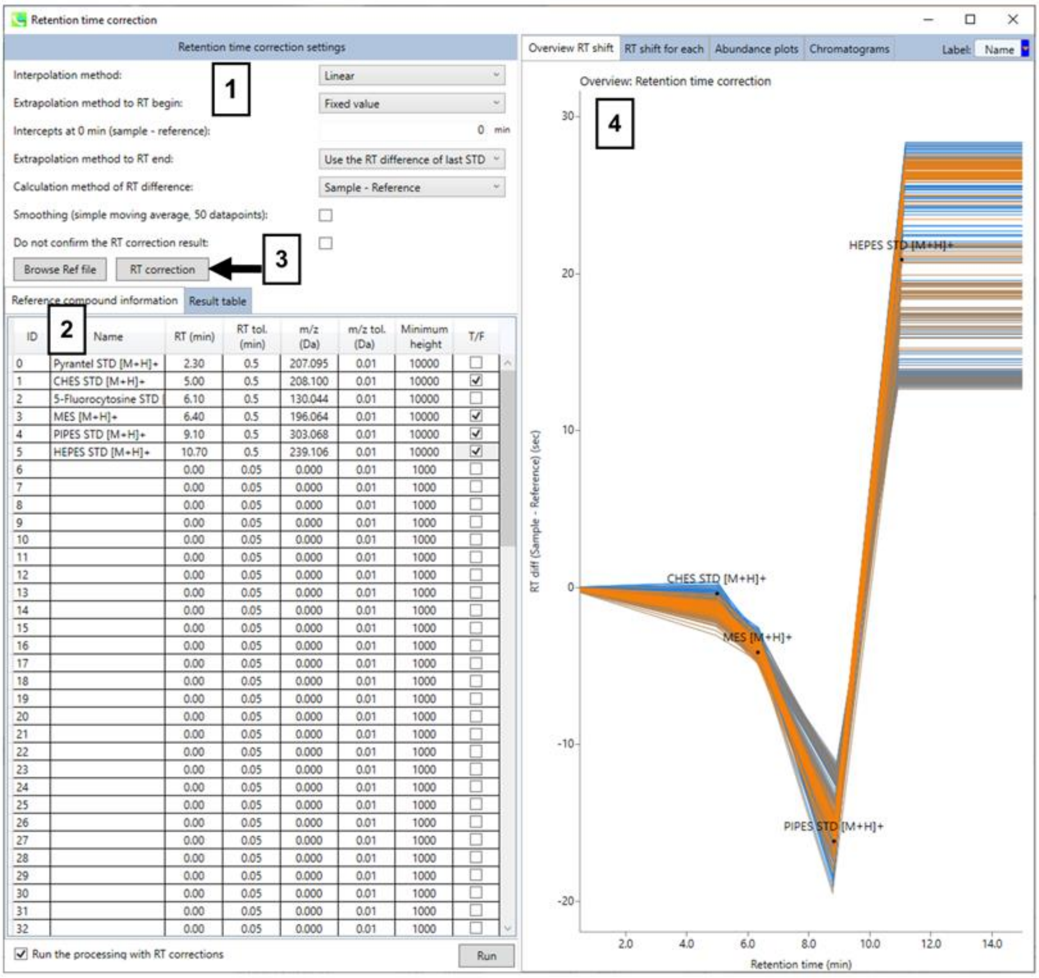

Figure 12. RT correction setting and overview. (1) set the values, (2) paste and confirm the information on the peaks for RT correction, (3) press “RT correction” to obtain, and (4) overview of the RT shifts.

Further information on the RT corrections can be obtained the “RT shift for each file”, “Abundance plots”, and “Chromatograms” tabs of the RT correction window (Figure 12). After deciding on the RT correction results, press the “Run” button (Figure 12) to proceed with data processing.

NOTE: Selection of values for data processing and RT correction parameters can require multiple trials until acceptable results are obtained. Depending on parameters, number of files and computer speed, the MS-DIAL data processing (peak detection, MS2Dec deconvolution, identification, alignment) can take several days or more for larger datasets.

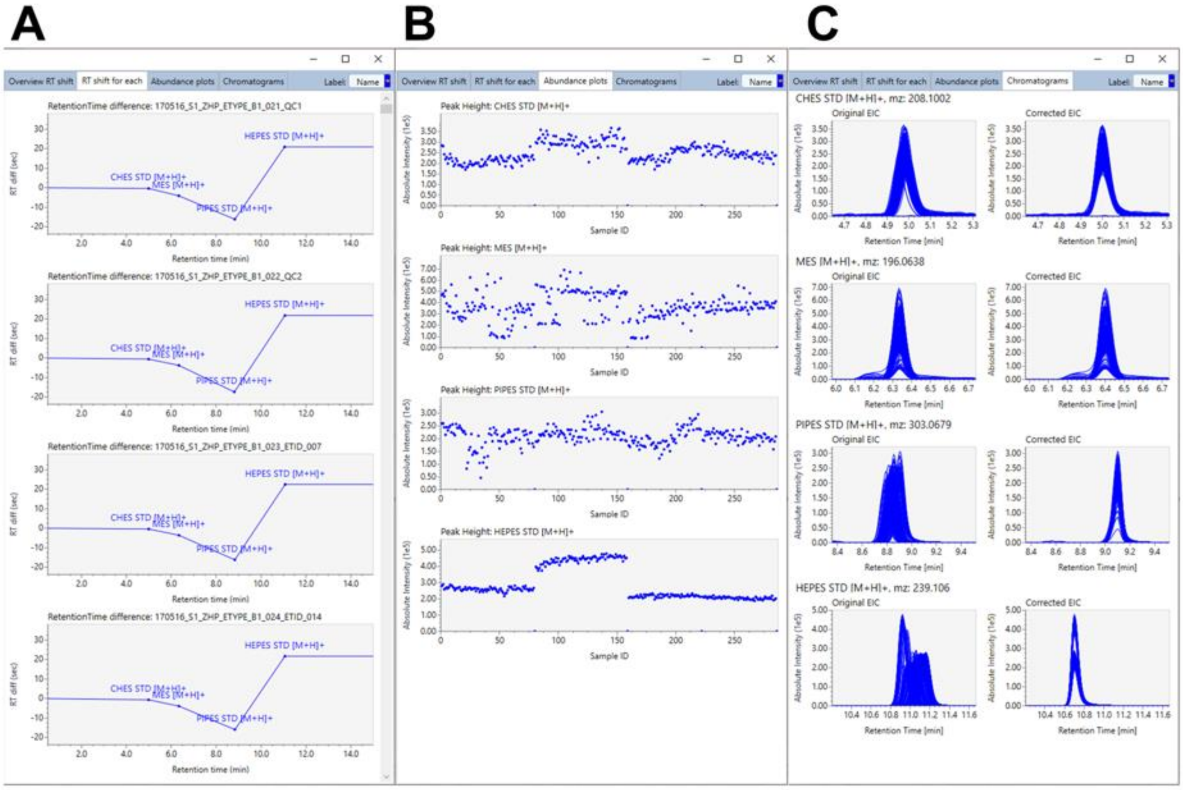

Figure 13. Details of the RT correction results: (A) RT shift for each file, (B) abundance plots, and (C) chromatograms.

4. Reviewing compound annotations

In MS-DIAL, the alignment and identification results depend on multiple parameters. Furthermore, thresholds and scoring of the compound identification matches are contentious topics. Therefore, in this tutorial we focus on the usage of AMRT+MS2 library for AIF data and provide several metabolite identification examples. Namely, early eluting pantothenic acid (Figure 14), relatively late eluting acetyl-histidine (Figure 15), closely eluting N_2- and N_6-acetyl-lysines (Figure 16 and 17), low-abundant tryptophan betaine (Figure 18), variable and highly abundant trigonelline (Figure 19), as well as late-eluting HEPES (Figure 20). The identification results of these compounds are summarized in Table 1.

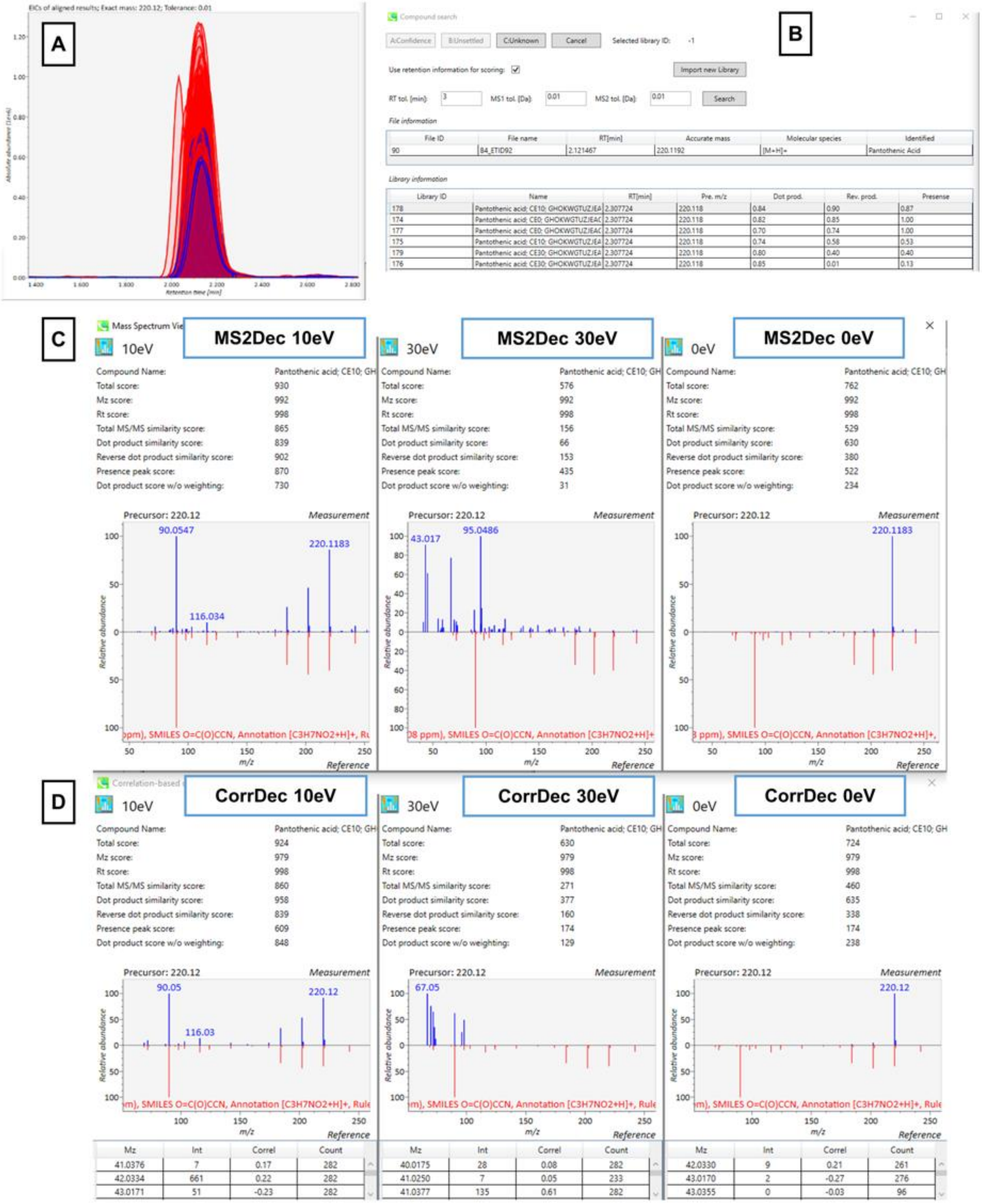

Figure 14. Identification of pantothenic acid. (A) Peak intensity is high (>1.2E6) and elution time early (2.13 min). (B) AMRT match in the library—pantothenic acid (RT shift –0.18 min (raw) or –0.17 min (RT corrected)). MS spectra deconvoluted by MS2Dec (C) and CorrDec (D) have high match scores at 10 eV, 930 and 924 respectively. In MS2Dec spectra, peaks above the precursor mass are matching.

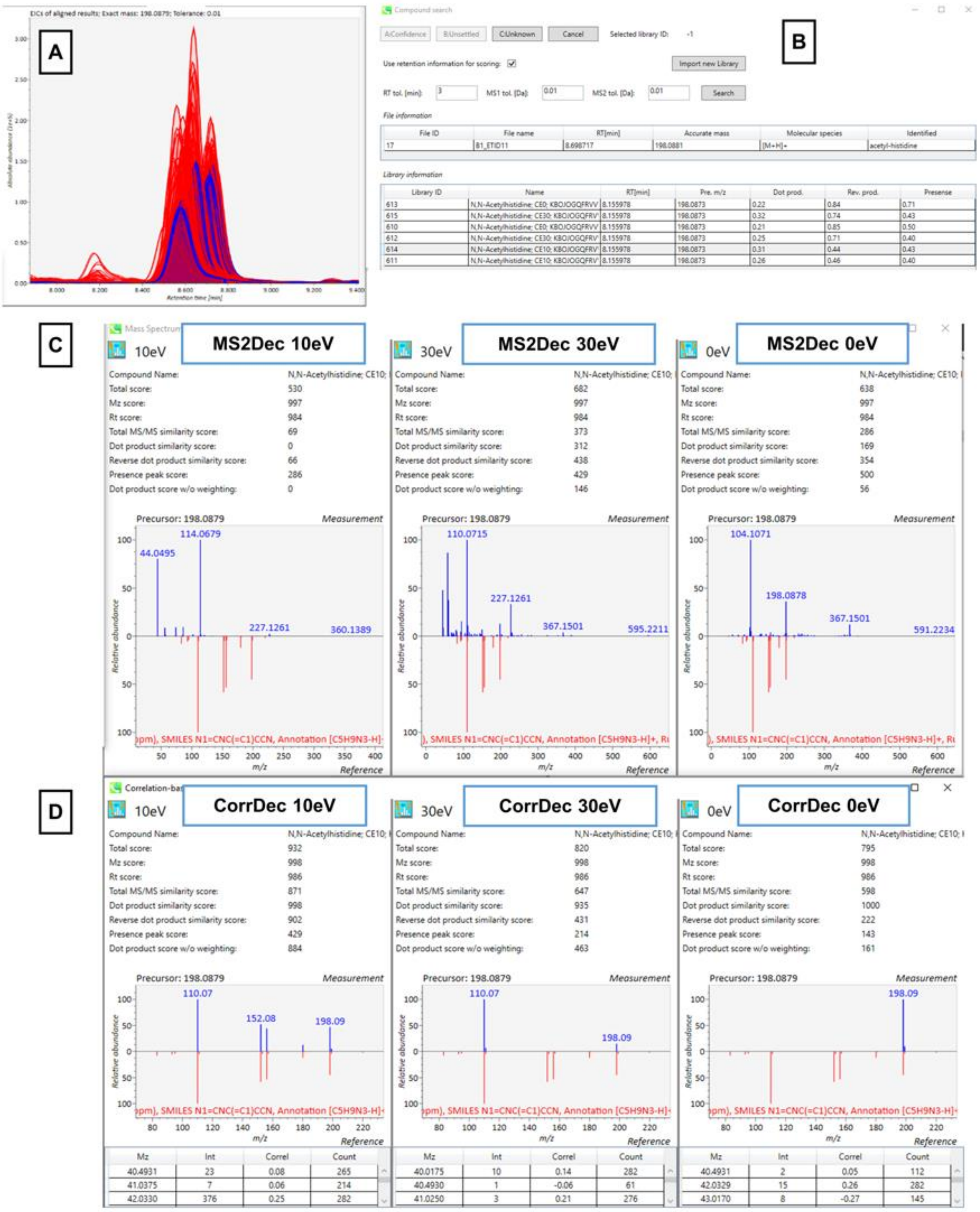

Figure 15. Identification of acetyl-histidine. (A) Peak intensity is medium (>3E5), elution time late and a bit fluctuating (8.66 min). (B) AMRT match in the library—N,N-acetyl-histidine acid (RT shift 0.50 min (raw) or 0.73 min (RT corrected)). Among MS spectra deconvoluted by MS2Dec (C) and CorrDec (D), MS2Dec spectra are rather noisy, but CorrDec spectra at 10 eV has high score (932) with five matching fragments.

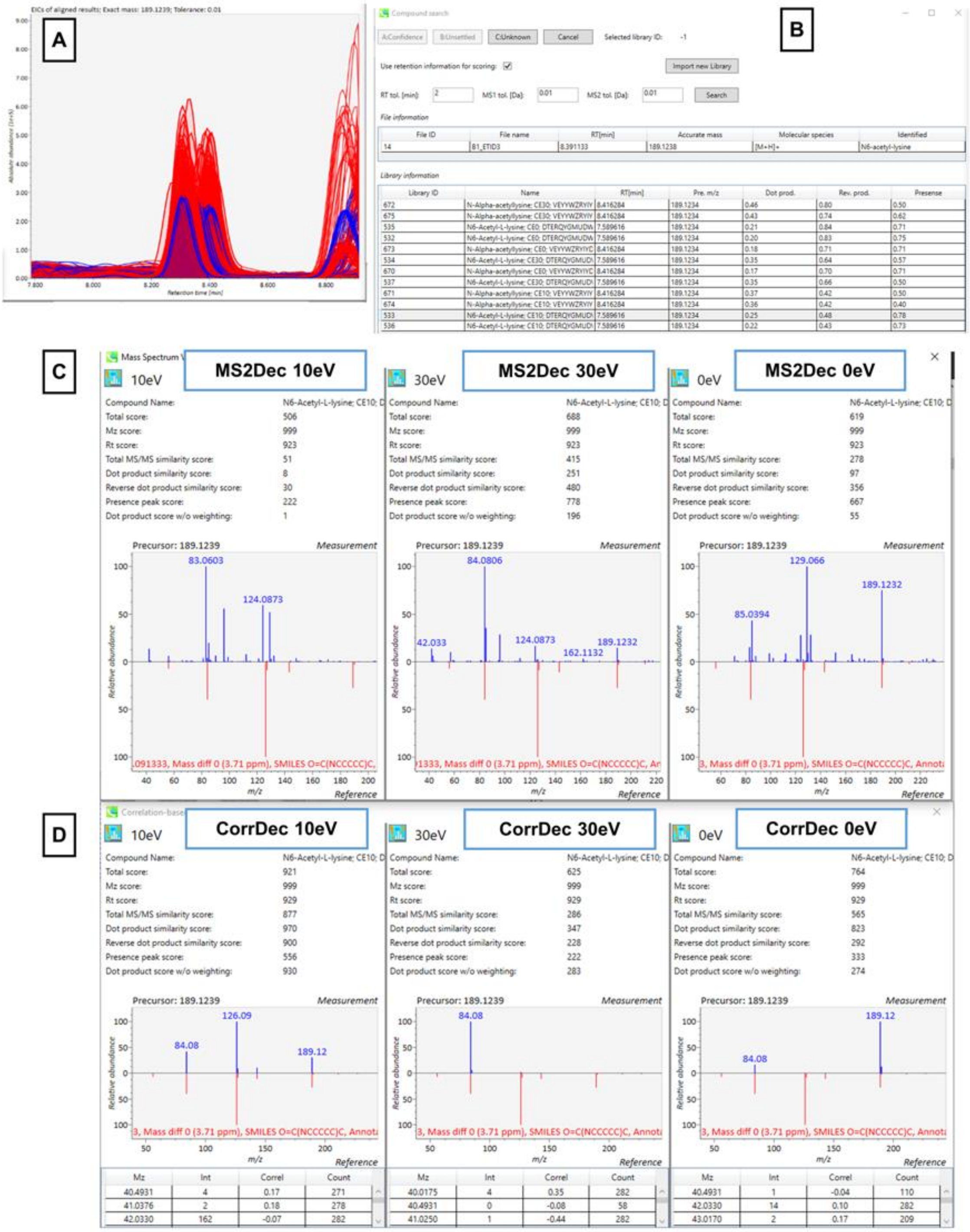

Figure 16. Identification of N_6-acetyl-lysine. (A) Peak intensity is medium (>6E5), two isobaric peaks eluting at 8.36 and 8.96 min. (B) AMRT matches in the library—acetyl-lysines, first eluting peak is N_6-acetyl-lysine (RT shift 0.77 min (raw) or 0.97 min (RT corrected)). Among MS spectra deconvoluted by MS2Dec (C) and CorrDec (D), CorrDec spectra at 10 eV has high score (921) with five matching peaks.

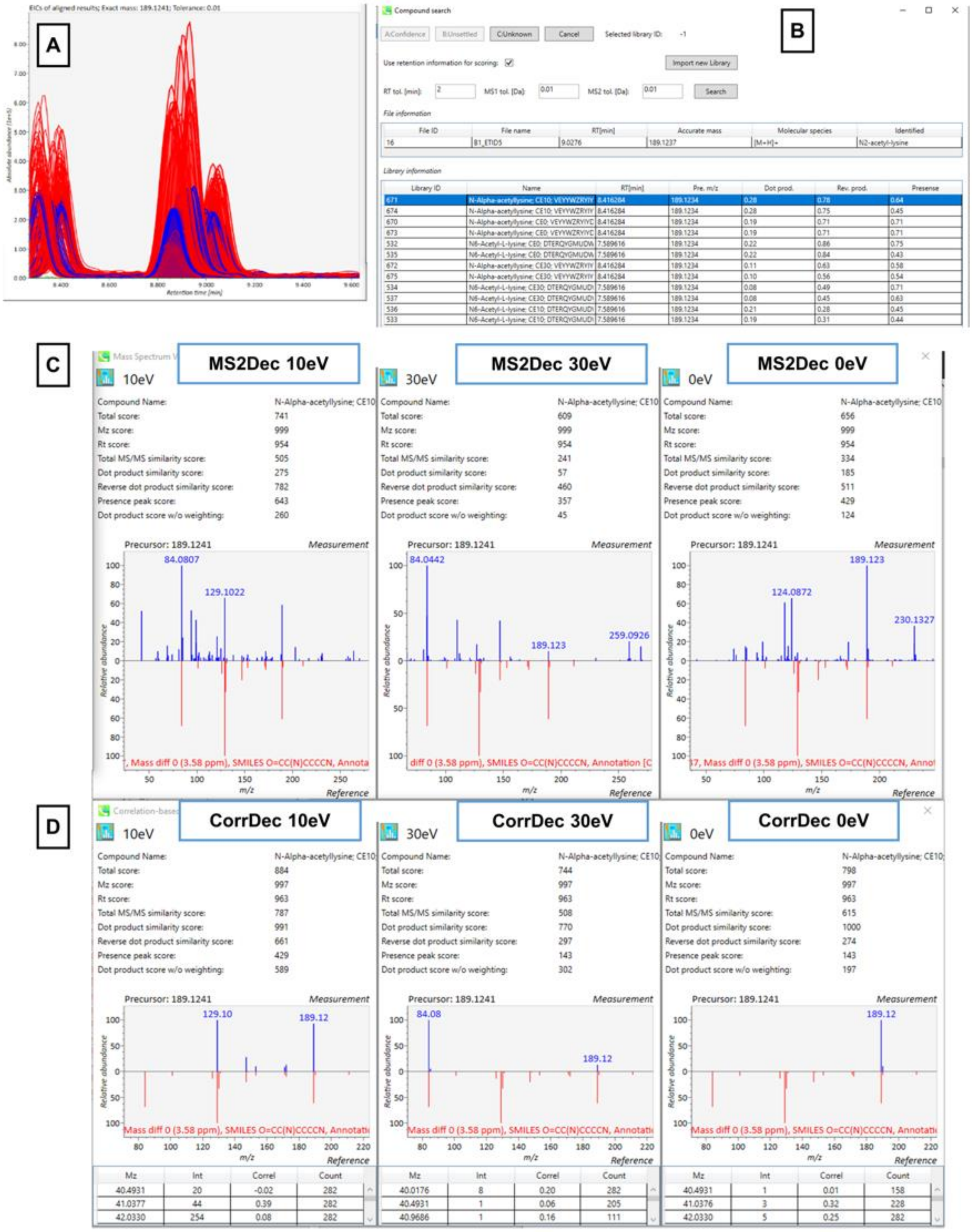

Figure 17. Identification of N2-acetyl-lysine. (A) Peak intensity is medium high (>8E5), two isobaric peaks eluting at 8.36 and 8.96 min. (B) AMRT matches in the library—acetyl-lysines, second eluting peak is N2-acetyl-lysine (RT shift 0.54 min (raw) or 0.75 min (RT corrected)). Among MS spectra deconvoluted by MS2Dec (C) and CorrDec (D), CorrDec spectra at 10 eV has high score (884) with five matching peaks.

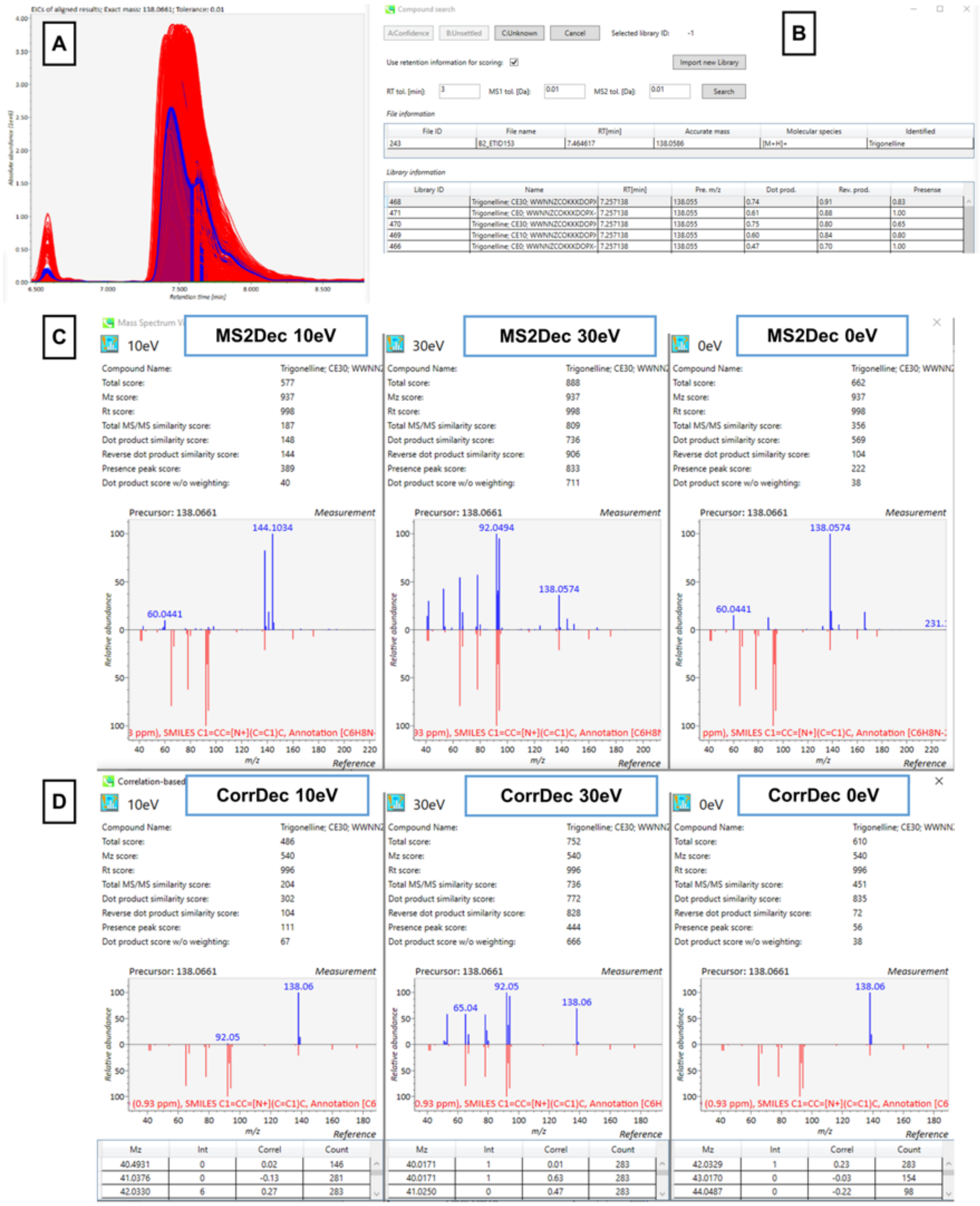

Figure 18. Identification of trigonelline. (A) Peak intensity is very high (>3E6), eluting at 7.5 min. (B) AMRT matches in the library—trigonelline (RT shift 0.25 min (raw) or 0.40 (RT corrected)). Among MS spectra deconvoluted by MS2Dec (C) and CorrDec (D), MS2Dec spectra at 30 eV has high score (888) with several matching peaks.

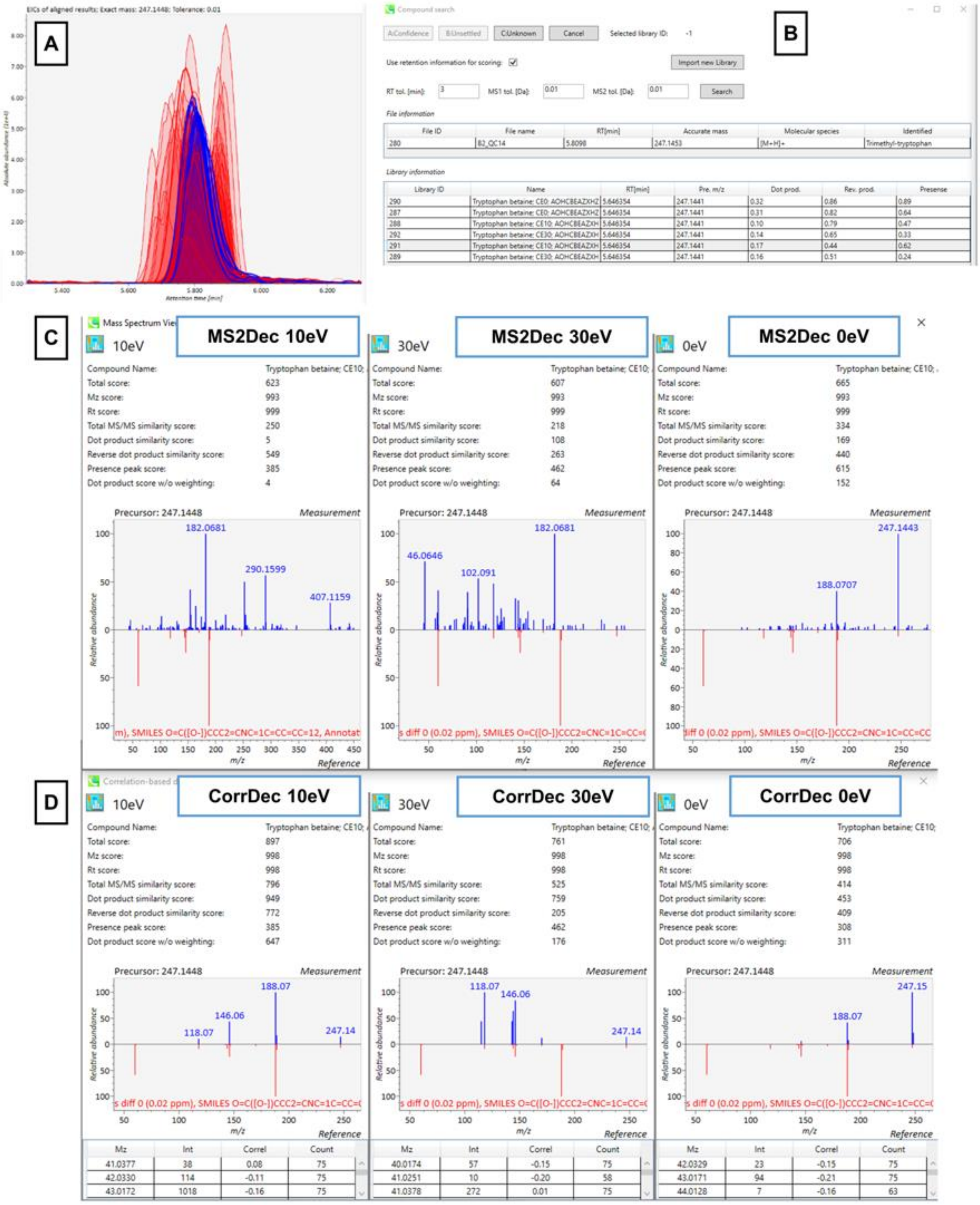

Figure 19. Identification of tryptophan betaine. (A) Peak intensity is low (>8E4), eluting at 5.82 min. (B) AMRT match in the library—tryptophan betaine (RT shift 0.17 min (raw) or 0.20 (RT corrected)). Among MS spectra deconvoluted by MS2Dec (C) and CorrDec (D), MS2Dec spectra are noisy, while CorrDec at 10eV has high score (897) with several matching peaks.

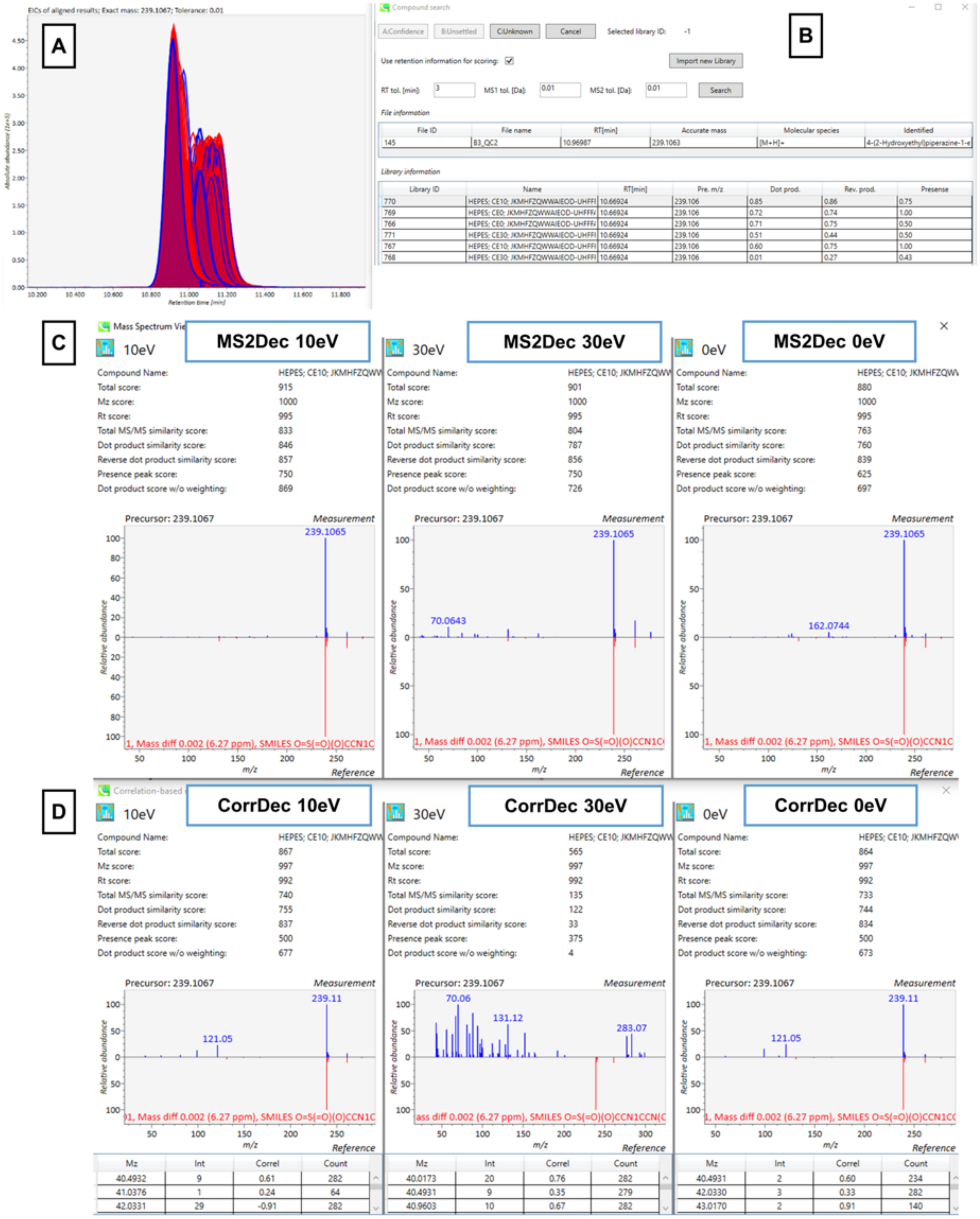

Figure 20. Identification of HEPES (tIS spiked during sample preparation in all samples). (A) Peak intensity is medium (>4E5), eluting at 11.05 min. (B) AMRT matched in the library—HEPES (RT shift 0.38 min (raw) or 0.03 (RT corrected)). Among MS spectra deconvoluted by MS2Dec (C) and CorrDec (D), MS2Dec spectra shows good matches (915 at 10 eV), while CorrDec spectra are noisy due to very similar levels of HEPES in the samples. In MS2Dec and CorrDec spectra, peaks above the precursor mass are matching.

Table 1. Summary of compound identification parameters

Compound	m/z library	m/z detected	ppm error	Max MS1 intensity	RT library (min)	RT raw / RT corrected	RT shift raw/ corrected	MS2 spectrum/ score	Comment
Pantothenic acid	220.1180	220.1200	9.1	1.2E6 (high)	2.31	2.13/ 2.14	-0.18/ -0.17	MS2Dec 10 eV/ 930	higher mass ppm possibly due to high intensity
Acetyl-histidine	198.0873	198.0879	3.0	3E5 (medium)	8.16	8.66/ 8.89	0.50/ 0.73	CorrDec 10 eV/ 932	similar RT shift as other compounds eluting in the region
N_6-acetyl-lysine	189.1234	189.1239	2.6	3E5 (medium)	7.59	8.36/ 8.57	0.77/ 0.97	CorrDec 10 eV/ 921	two closely eluting isobaric peaks, high RT shift
N_2-acetyl-lysine	189.1234	189.1241	3.7	8E5 (medium)	8.42	8.96/ 9.17	0.54/ 0.75	CorrDec 10 eV/ 884	two closely eluting isobaric peaks
Trigonelline	138.0550	138.0661	80.4	3E6 (high)	7.26	7.51/ 7.66	0.25/ 0.40	MS2Dec 30 eV/ 888	very high mass ppm due to high intensity
Tryptophan betaine	247.1441	247.1448	2.8	8E4 (low)	5.65	5.82/ 5.85	0.17/ 0.20	CorrDec 10 eV/ 897	low abundant, MS2Dec spectra noisy
HEPES	239.1060	239.1067	2.9	4E5 (medium)	10.67	11.05/ 10.70	0.38/ 0.03	MS2Dec 10 eV/ 915	tIS, CorrDec deconvolution poor due to similar levels in the samples

The summary of compound identification values in Table 1 shows that each parameter used in compound identification can be affected depending on specific compound characteristics in the samples. AM can drift as much as 80 ppm due to the high abundances, as in the case of trigonelline. RT shifts are complex due to multiple factors (e.g., column batches, matrix effects etc.). From the provided examples, it can be seen that RT corrections do not always work. Especially around 8–9 min, the corrected RT shift of the tIS PIPES seems to be opposite to that of the example metabolites. It should be noted that the library is not matrix-specific, and around 8 min is the elution time of creatinine, a very high abundant urinary metabolite that can create RT interferences. Finally, while all of the example compounds have at least one good spectral match, it is clear from Figures 14 to 20 that results can very much differ between deconvolution method and collision energy. For example, CorrDec deconvolution works relatively well for low-abundant variable compounds (tryptophan betaine), while MS2Dec works better if the compound is less variable among the samples and not low-abundant (HEPES). However, while each parameter can be affected, the combination of multiple parameters provides a higher level on confidence in metabolite identification. Therefore, it is import to understand what are the limitations and properties of the instrumentation/method (e.g., AM and RT shifts) as well as MS spectra (e.g., deconvolution method) to provide reliable and transparent identifications for the AIF datasets.

5. <u>Notes</u>

- Distinction of structurally similar co-eluting metabolites (e.g., dimethylxanthines in current method) requires a different approach, as detailed here. Specific fragment for each co-eluting metabolite needs to be identified (e.g., Chaleckis et al 2018, PMID: 29363064). However, such fragment might not exist or in too low intensities/abundances in the study samples. Metabolite name and identification level should be adapted accordingly.
- A matrix-specific AMRT+MS2 library constructed from AIF data might provide better spectral matches as it will include eventual matrix-specific RT shifts as well as matrix-specific adducts.
- Including more and different peaks for RT correction might reduce the RT drifts, however, especially in HILIC chromatography, the shifts are complex, with groups of compounds drifting into opposite directions. While the tIS are often essential to manage RT drifts, the importance of RT accuracy also depends on the LC method and metabolites of interest (e.g., hydrophilic metabolites or lipids), composition and complexity of the sample (plasma/serum, urine, cellular extract etc.), and "peak purity" of metabolites in extracted ion chromatogram (EIC). For example, in conventional untargeted lipidomics, multiple closely eluting isobaric peaks can only be confidently annotated (e.g., acyl chain compositions and positions *sn*1/*sn*2/*sn*3 positional isomers) if confirmed by internal standards analyzed in the same analytical batch. On the other hand, if only a single peak is detected in the entire EIC of the metabolite of interest, then the accurate RT is less critical. Also, the composition of the sample (e.g., cellular extract vs. urine) plays a role in the RT shifts due to the matrix effects.
