# Supplementary figures and images for: Creating a Reliable Mass Spectral–Retention Time Library for All Ion Fragmentation-Based Metabolomics
Source: Metabolites. 2019 Oct 26;9(11):251. doi: 10.3390/metabo9110251 (PMC6918128; doi:10.3390/metabo9110251)

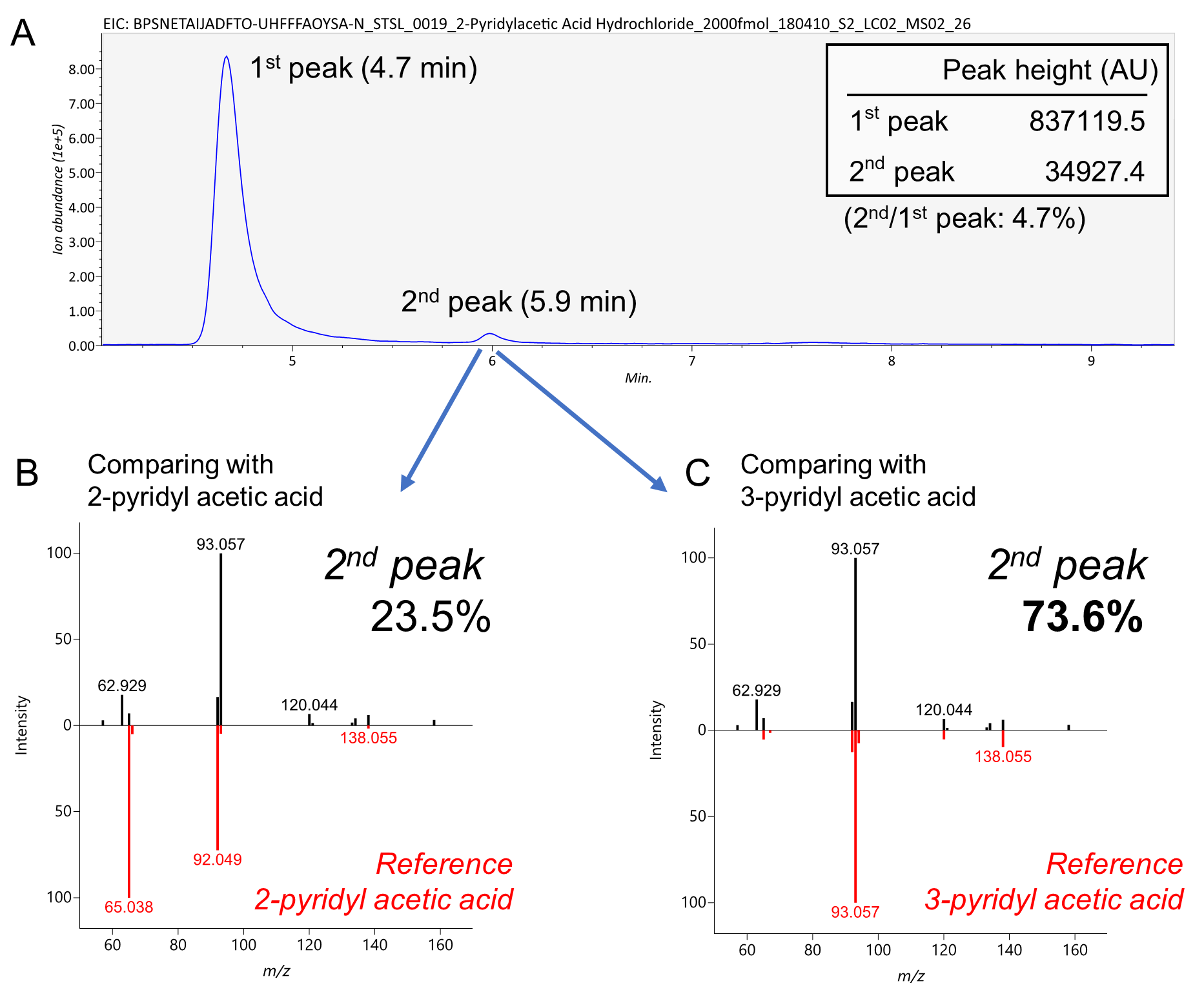

Supplement: Supplementary file 1 [file metabolites-09-00251-s001.zip › supplementary materials/Figure S1.tif]

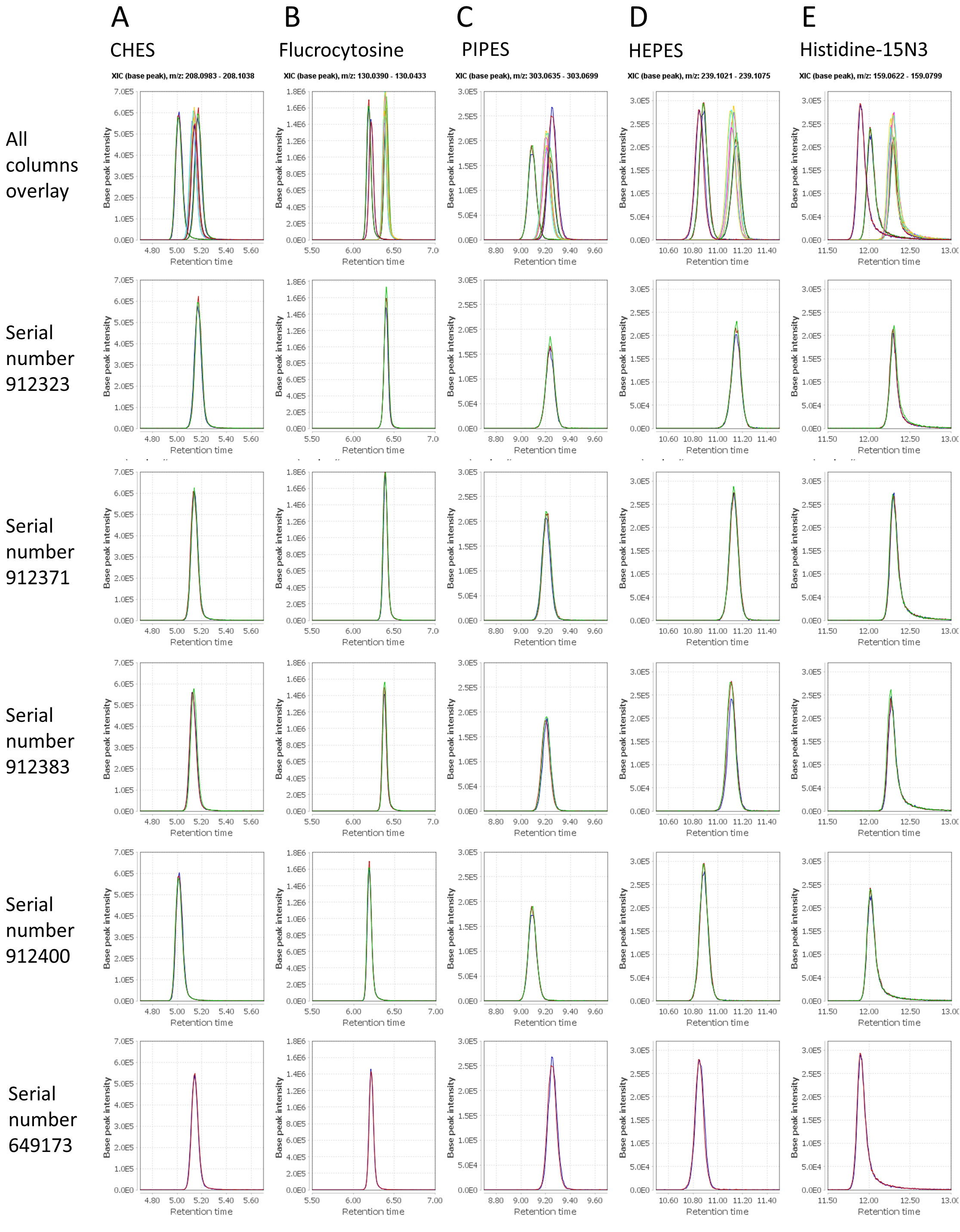

Supplement: Supplementary file 1 [file metabolites-09-00251-s001.zip › supplementary materials/Figure S2.tif]

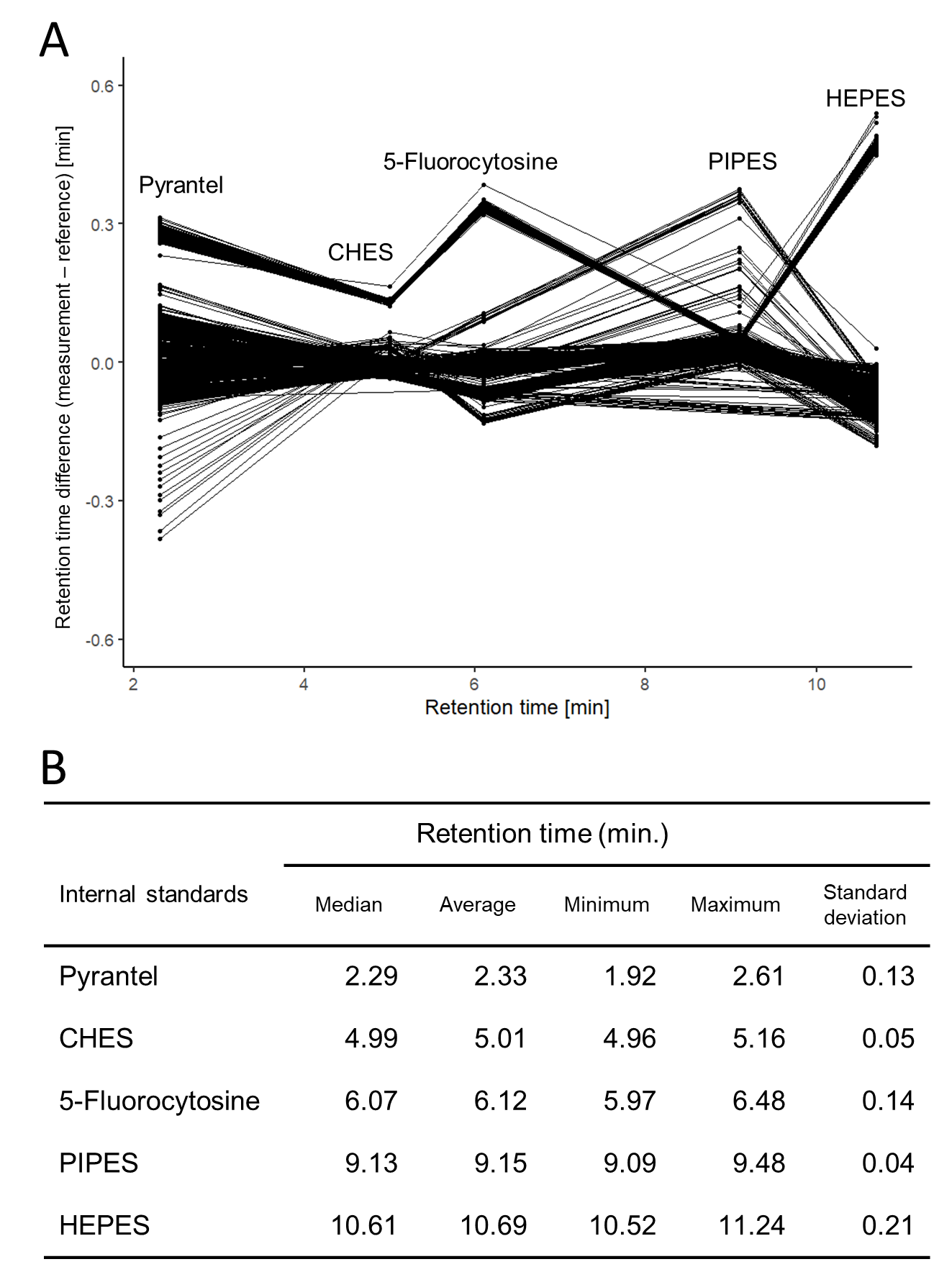

Supplement: Supplementary file 1 [file metabolites-09-00251-s001.zip › supplementary materials/Figure S3.tif]
